# A non-canonical JAK/STAT pathway promotes viral replication through the lipoprotein receptor-related protein in ticks

**DOI:** 10.1371/journal.pbio.3003797

**Published:** 2026-05-21

**Authors:** Yan Xu, Wenbo Zeng, Guodian Xiong, Zhenhua Zheng, Jingwen Wang

**Affiliations:** 1 State Key Laboratory of Genetics and Development of Complex Phenotypes, Department of Infectious Diseases, Fudan University, School of Life Sciences, Zhongshan Hospital, Fudan University, Shanghai, China; 2 Ministry of Education Key Laboratory of Contemporary Anthropology, School of Life Sciences, Fudan University, Shanghai, China; 3 Center for Emerging Infectious Diseases, Wuhan Institute of Virology, Chinese Academy of Sciences, Wuhan, Hubei, China; Duke-NUS Medical School, SINGAPORE

## Abstract

Ticks transmit numerous viruses that pose significant threats to human and animal health, however, the molecular interactions between ticks and viruses remain poorly understood. Using Langat virus (LGTV)—a surrogate for tick-borne encephalitis virus (TBEV), we show that the JAK/STAT pathway promotes viral infection in *Haemaphysalis longicornis*. Rather than directly interacting with viral proteins, this proviral effect is mediated by a low-density lipoprotein receptor-related protein (LRP), whose expression is regulated by STAT. Silencing *LRP* in *H. longicornis* reduced LGTV infection, while ectopic expression of *LRP* enhanced it. Unlike its mammalian counterparts, *H. longicornis* LRP lacks a transmembrane domain and localizes intracellularly. Functionally, LRP promotes lipophagy, leading to lipid droplets breakdown and providing energy to support viral replication. Together, these findings reveal a non-canonical mechanism by which the JAK/STAT pathway facilitates LGTV replication through STAT-dependent regulation of an atypical, intracellular LRP that drives lipophagy.

## Abstract 2

Tick–virus interactions are poorly understood despite their impact on disease transmission. This study shows that ticks hijack the JAK/STAT signaling pathway to drive LRP-mediated lipophagy, supplying energy that enhances viral replication.

## Introduction

Ticks are hematophagous arthropods that serve as vectors for a wide array of pathogens, including viruses, bacteria, and protozoa, with certain species capable of transmitting multiple agents simultaneously [1]. These pathogens are responsible for numerous diseases in humans and animals, such as tick-borne encephalitis, Crimean-Congo hemorrhagic fever, Lyme disease, and babesiosis. Among these, tick-borne flaviviruses pose a significant threat to public and veterinary health and are associated with substantial economic impact [2]. Key members of this group include tick-borne encephalitis virus (TBEV), Powassan virus (POWV), Kyasanur Forest disease virus (KFDV), and Alongshan virus (ALSV).

TBEV is one of the most important human tick-borne flaviviruses, capable of causing severe neurological complications or death. Notably, its incidence has risen in recent years. There are three main subtypes of TBEV: European (TBEV-Eu), Siberian (TBEV-Sib), and Far Eastern (TBEV-FE). TBEV-Eu infections are typically mild and rarely result in serious sequelae, with a reported mortality rate of 0.5%–2%. In contrast, TBEV-Sib infections are generally less symptomatic but are more likely to become prolonged, with a mortality rate of 2%–3%. TBEV-FE infections are the most severe, with mortality rates reaching up to 40% [3,4]. TBEV-Eu is mainly transmitted by *Ixodes ricinus*, while TBEV-Sib and TBEV-FE are primarily transmitted by *Ixodes persulcatus* [5]. Due to its classification as a biosafety level 3 (BSL-3) pathogen, research on TBEV is constrained, and most studies have relied on mammalian cell lines. Consequently, the mechanisms underlying TBEV maintenance and transmission in tick vectors remain poorly understood. Langat virus (LGTV), a naturally attenuated tick-borne flavivirus sharing 82%–88% amino acid identity with TBEV, is widely used as a surrogate model for TBEV in research [6]. We have previously established a LGTV–tick–mouse transmission model and demonstrated that *H. longicornis* is a competent vector for LGTV [7]. However, the molecular interactions between *H. longicornis* and LGTV remain largely unexplored.

Ticks rely on their innate immune system to combat pathogen infections [8,9]. The Janus kinase/signal transducer and activator of transcription (JAK/STAT) signaling pathway is one of the classical immune signaling pathways that tick harbor and is involved in both pathogen infection and development in ticks. In *Ixodes scapularis*, for instance, the JAK/STAT pathway mediates defense against *Anaplasma phagocytophilum* by regulating the expression of 5.3-kDa antimicrobial peptides [10]. Conversely, it facilitates colonization by *Borrelia burgdorferi* by maintaining the integrity of the peritrophic matrix [11]. In addition, JAK/STAT pathway in *I. scapularis* can be activated by host-derived IFN-γ, thereby accelerating blood ingestion and development [12]. Despite its established roles in bacterial defense and tick physiology, the function of the JAK/STAT pathway in antiviral responses in ticks remains largely unexplored.

In this study, we demonstrate that the JAK/STAT signaling pathway facilitates LGTV infection in ticks by regulating the expression of lipoprotein receptor-related protein (LRP). LRP is a host factor that promotes the degradation of lipid droplets (LDs) through lipophagy, thereby releasing lipids that are essential for viral replication.

## Results

### JAK/STAT pathway facilitates LGTV infection in *H. longicornis*

**Fig 1:**
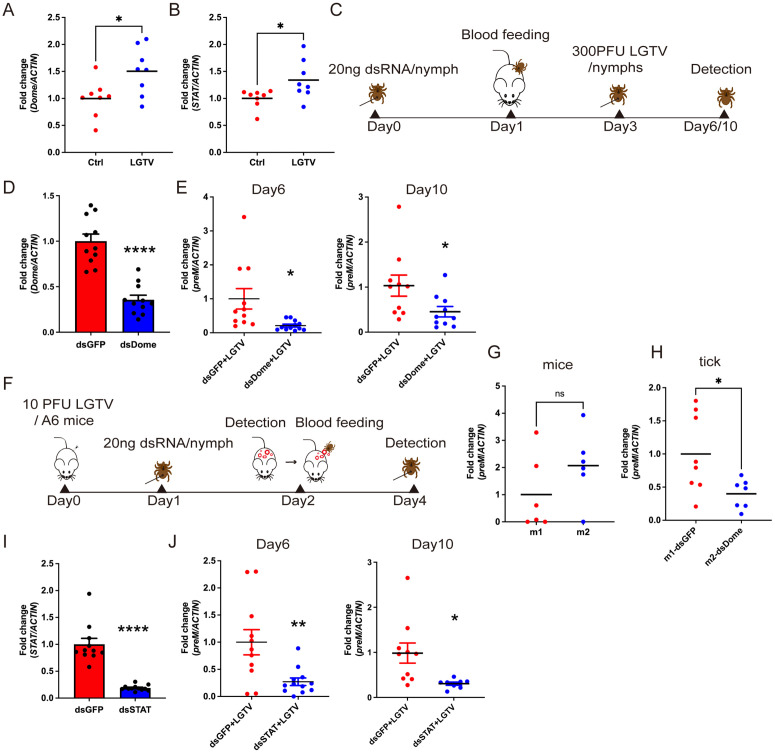
Knockdown of JAK/STAT pathway inhibits LGTV infection. (**A**, **B**) Quantification of *Dome* and *STAT* gene levels in LGTV-infected nymphs. (**C**) Schematic of experimental design for dsRNA treatment and LGTV infection via injection. (**D**, **E**) Quantification of *Dome* knocking down efficiency (*D*) and *preM* levels (*E*) by qPCR in dsDome and dsGFP nymphs. (**F**) Schematic of experimental design for dsRNA treatment and LGTV infection via blood feeding. (**G**, **H**) Quantification of *preM* gene levels in mice (*G*) and dsRNA-treated nymphs (H). (**I**, **J**) Quantification of *STAT* knocking down efficiency (*I*) and *preM* levels (*J*) by qPCR in dsSTAT and dsGFP nymphs. Each dot represents 2 pooled nymphs in (A), (B), (D), (E), (H), (I), (J), and individual mouse blood sample in (G). Data are presented as mean ± SEM in (D) (*n* = 11) and (I) (*n* = 11–12). Horizontal lines represent the mean in (A) (*n* = 8), (B) (*n* = 8), (E) (*n* = 10–12), (G) (*n* = 6), (H) (*n* = 7–8), and (J) (*n* = 10–11). Significance was determined by Student *t* test in (A), (B), (D), (E), (G), (H), (I), and (J). * *p* < 0.05, ** *p* < 0.01, *** *p* < 0.001, **** *p* < 0.0001. The data underlying this figure are provided in S1 Raw Data.

To investigate the role of JAK/STAT pathway during LGTV infection in *H. longicornis*, we first assessed the expression profiles of the receptor, *domeless* (*Dome*) and transcription factor (*STAT*), and found significant upregulation of both genes in nymphs at two days post-feeding on LGTV-infected mice (Fig 1A and 1B). We next knocked down the receptor, *domeless* (*Dome*), in unfed nymphs. After a two-day feeding period, the nymphs were infected with LGTV via microinjection, and viral load was quantified by measuring the expression of the LGTV membrane-associated glycoprotein precursor (*preM*) gene at day 6 (3 days post-infection, dpi) and day 10 (7 dpi) (Fig 1C). Surprisingly, *Dome* knockdown significantly reduced LGTV infection compared to the dsGFP control at both time points (Fig 1D and 1E). To mimic natural virus acquisition, we next allowed dsRNA-treated nymphs to feed on LGTV-infected A6 mice, which are deficient in the type I interferon (IFN) receptors (A6 mice) (Fig 1F). To ensure that the mice used for tick feeding exhibit similar viremia, we measured viremia in six infected mice at 2 dpi and selected two with similar viral titers for tick feeding (Fig 1G). After two days of feeding, ticks were collected, and viral loads were assessed. Consistent with the injection-based results, Dome knockdown significantly reduced LGTV infection compared to controls (Fig 1H). To further confirm the involvement of the JAK/STAT pathway, we knocked down *STAT*, the downstream transcription factor, and analyzed infection outcomes. As expected, *STAT* knockdown also significantly inhibited LGTV replication at both day 6 and day 10 (Fig 1I and 1J). Given that *STAT* silencing also impairs blood feeding in *H. longicornis*, as previously observed in *Ixodes* ticks (S1 Fig) [12], we used microinjection for subsequent infections to ensure equal viral input across treatments. Moreover, knockdown of *Dome* or *STAT* in *I. scapularis* ISE6 cells similarly suppressed LGTV infection (S2 Fig), suggesting a conserved pro-viral role of the JAK/STAT pathway across tick species.

### JAK/STAT pathway promotes LGTV infection via regulating LRP expression

Viruses often manipulate the JAK/STAT pathway by targeting STAT proteins, particularly through interactions with viral non-structural (NS) proteins that interfere with STAT phosphorylation or nuclear translocation [13,14]. To test whether LGTV employs a similar strategy, we investigated potential interactions between STAT and LGTV NS proteins. HEK-293T cells were co-transfected with plasmids expressing STAT tagged with a Flag tag (STAT-Flag) and individual HA-tagged LGTV NS proteins (NS1–HA, NS2A–HA, NS2B–HA, NS3–HA, NS4A–HA, NS4B–HA, and NS5–HA). Co-immunoprecipitation was performed using anti-Flag beads to pull down STAT and any associated proteins. Surprisingly, none of the NS proteins co-precipitated with STAT, indicating the absence of direct interactions between STAT and LGTV NS proteins (S3 Fig).

**Fig 2:**
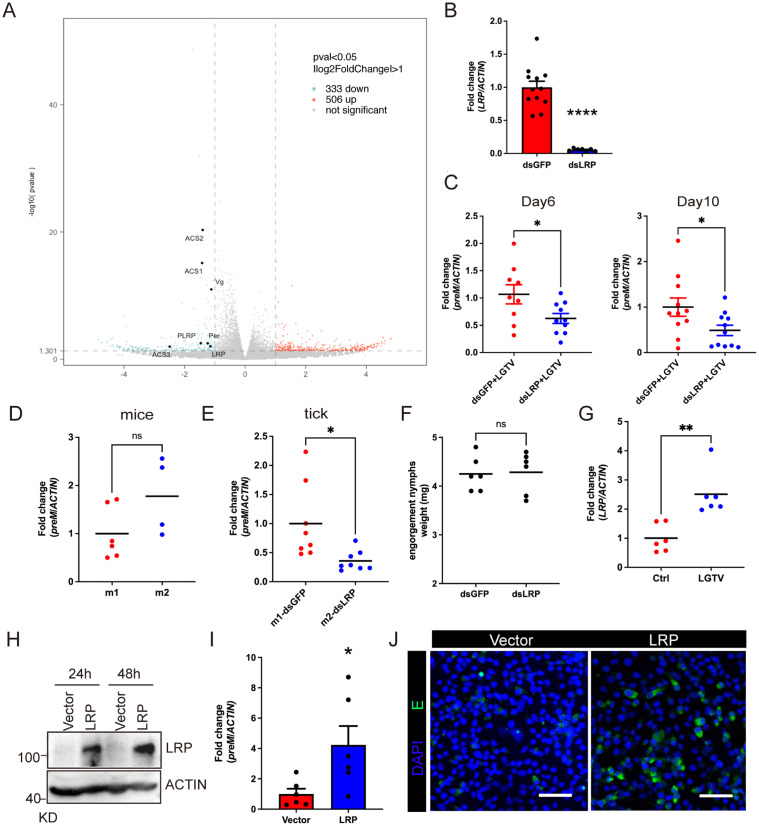
LRP promotes LGTV infection. (**A**)The volcano plot of differentially expressed genes in dsGFP and dsSTAT ticks. Red circles represent 506 significantly up-regulated genes, and green circles represent 333 significantly down-regulated genes (Padj < 0.05). All genes showing significant changes were listed in the S1 Table. (**B**, **C**) Quantification of *LRP* knocking down efficiency (*B*) and *preM* levels (*C*) by qPCR in dsLRP and dsGFP nymphs. (**D**, **E**) Quantification of *preM* gene levels in mice and dsRNA-treated nymphs. (**F**) The 2-day feeding weights of nymphs treated with dsGFP and dsLRP. (**G**) Quantification of *LRP* gene levels in LGTV-infected nymphs. (**H**) Verification of LRP expression in BHK-21 cells by western blot analysis. The pCAGGS vector served as control. (**I**, **J**) Influence of LRP overexpression on LGTV infection in BHK-21 cells. The levels of *preM* and E protein were accessed by qPCR (*I*) and immunohistochemistry (J), respectively. Cells were stained with anti-EDⅢ antibody (green). Nuclei were stained with DAPI (blue). Scale bar: 50 μm. Each dot represents 2 pooled nymphs in (B), (C), (E), (G), 10 nymphs in (F), individual mouse blood sample in (D), and individual cell well in (I). Data are presented as mean ± SEM in (B) (*n* = 7–12) and (I) (*n* = 6). Horizontal lines represent the mean in (C) (*n* = 10–11), (D) (*n* = 4–6), (E) (*n* = 8), (F) (*n* = 6), and (G) (*n* = 6). Significance was determined by Student *t t*est in (B), (C), (D), (E), (F), (G), and (I). * *p* < 0.05, ** *p* < 0.01. The data underlying this figure are provided in S1 Raw Data. The uncropped blots are included in S1 Raw Images.

To investigate the mechanism of JAK/STAT-mediated promotion of LGTV infection, we next compared the gene expression pattern between dsGFP- and dsSTAT-treated ticks via RNA sequencing. A total of 839 genes were differentially regulated, with 506 upregulated and 333 downregulated (Fig 2A and S1 Table). Among the downregulated genes were several known to facilitate viral infections, including members of the low-density lipoprotein receptor family (*LRP*, *PLRP*), vitellogenin (*Vg*), peritrophin (*Per*), and acyl-coenzyme A synthetases (*ACS1*, *ACS2*, *ACS3*) [15–18]. To explore whether these genes also contribute to viral infection in *H. longicornis*, we picked up 4 downregulated genes in dsSTAT ticks and examined their roles in LGTV infection. They were *LRP*, *ACS1*, *Vg*, and *Per*. Interestingly, only *LRP* knockdown significantly reduced viremia of LGTV compared to the dsGFP groups on both day 6 and day 10 (Figs 2B, 2C, and S4A). No difference in infection rate was observed in the other knockdown groups (S4B–S4D Fig). We next simulated the natural virus acquisition process in ticks described in Fig 1F. Despite comparable blood meal amount at 2 days post-attachment, *LRP*-deficient nymphs showed significantly lower LGTV loads than dsGFP-treated controls (Fig 2D–2F). Consistent with the expression patterns of *Dome* and *STAT*, LGTV infection triggered a significant upregulation of *LRP* (Fig 2G). Collectively, these results demonstrate that LRP, regulated via the JAK/STAT pathway, facilitates LGTV infection in tick nymphs.

To validate the role of LRP in promoting LGTV infection, we next ectopically expressed Flag-tagged LRP (LRP-Flag) in BHK-21 cells, which are permissive to LGTV infection. At 24 hours post-transfection, the cells were infected with LGTV at a multiplicity of infection (MOI) of 1. Ectopic expression of LRP significantly enhanced LGTV replication, as evidenced by increased levels of *preM* mRNA and E protein expression (Fig 2H–2J). Together, these results support the conclusion that LRP is regulated via the JAK/STAT pathway and functions as a critical host factor that facilitates LGTV infection in tick nymphs.

### LRP is essential for LGTV replication

The LDLR family serves as the entry receptors for multiple alphaviruses and orthonairoviruses [15,16,19–21]. Members of this family are evolutionarily conserved in vertebrates and share the same structural features, including a signal peptide, a ligand binding domain (LBD) containing multiple LDLR type A (LDLa) repeats, epidermal growth factor (EGF)-like domains interspersed with a YWTD β-propeller (LY) module, a transmembrane anchor, and a cytoplasmic tail [22]. To assess the structural similarity between human LDLR and LRP across tick species, we analyzed the structure of LRP using the simple modular architecture research tool (SMART). Unlike human LDLR, *H. longicornis* LRP regulated by STAT only contains LDLa and EGF domains and lacks the LY module and transmembrane domain. Notably, LRP homologs lacking transmembrane domains were also identified in other tick species, including *Dermacentor silvarum*, *Ixodes persulcatus*, *Ixodes pacificus*, and *Ixodes scapularis* (S5A Fig). These results indicates that this atypical intracellular LRP architecture is conserved across diverse tick taxa.

Although structural analysis suggested that LRP is a cytosolic protein, we still assessed whether it could function as a receptor for LGTV, similar to classical LDLR family members in mammals. We investigated potential interactions between LRP and the LGTV envelope (E) protein in HEK-293T cells. Co-immunoprecipitation assays revealed no direct interaction between LRP and the E protein (S5B Fig). We also performed immunofluorescence analysis using confocal microscopy to examine the subcellular localization of LRP and the E protein in BHK-21 cells. Consistent with structural predictions, LRP was mainly localized in the cytoplasm, and no co-localization with the E protein was observed (S5C Fig). Furthermore, virus binding and internalization assays showed that ectopic expression of LRP had no significant impact on LGTV attachment or entry into host cells (S5D Fig). Together, these results indicate that LRP does not function as an entry receptor for LGTV.

**Fig 3:**
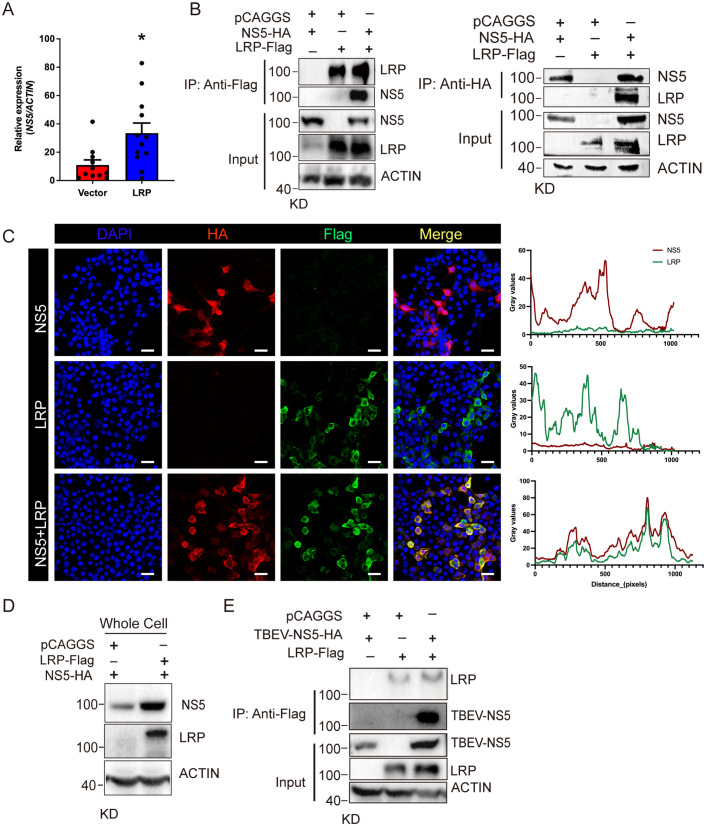
LRP promotes LGTV replication. (**A**) Quantification of *NS5* levels in LRP-expressed BHK-21 cells. Cells transfected with pCAGGS vector were served as control. (**B**) Co-immunoprecipitation of LRP-Flag and NS5-HA. The cell lysates were harvested and immunoprecipitated with antibodies against Flag (left) and HA (right). Samples were subjected to western blot analysis using antibodies against the Flag-tag, HA-tag, or ACTIN. (**C**) Co-localization (left) of LRP (green) with NS5 (red) in BHK-21 cells. Nuclei were stained by DAPI (blue). Scale bar: 25 μm. Quantification (right) was performed using at least 10 cells across three independent experiments. Pearson’s *R* = 0.90; Manders’ M1 = 0.92; M2 = 1.00; Costes’ P = 1.00. ****p < 0.001 by unpaired Student *t t*est. (**D**) The effect of LRP overexpression on NS5 in BHK-21 cells. The cell lysates were harvested for western blot analysis using antibodies against the Flag-tag, HA-tag, or ACTIN. (**E**) Co-immunoprecipitation of LRP and TBEV-NS5. The cell lysates were harvested and immunoprecipitated with antibodies against Flag. Samples were subjected to western blot analysis using antibodies against the Flag-tag, HA-tag, or ACTIN. Each dot represents individual cell well in (A). Data are presented as mean ± SEM in (A) (*n* = 11–12). Significance was determined by Student *t t*est in (A). * *p* < 0.05. The data underlying this figure are provided in S1 Raw Data. The uncropped blots are included in S1 Raw Images.

The cytoplasmic localization of LRP prompted us to hypothesize that it may play a role in viral replication. To test this, we transfected BHK-21 cells with LRP and introduced a LGTV replicon 24 hours later, thereby bypassing the viral entry step. After an additional 24 hours, we harvested the cells to quantify replicon levels. Compared to the control group transfected with the pCAGGS vector, LRP overexpression significantly increased *NS5* mRNA levels (Fig 3A), indicating that LRP enhances LGTV replication. Given NS5 encodes the RNA-dependent RNA polymerase (RdRp) essential for flavivirus genome replication [23,24], we next examined whether LRP exerts this effect through a physically interacts with NS5. We co-transfected HEK-293T cells with LRP-Flag and NS5-HA plasmids. Co-immunoprecipitation assays confirmed a direct interaction between LRP and NS5 (Fig 3B). Consistent with these results, confocal microscopy in BHK-21 cells showed that co-localization of NS5 and LRP (Fig 3C). In addition, the presence of LRP increased NS5 protein level (Fig 3D). Together, these findings suggest that LRP facilitates LGTV replication by interacting with NS5.

Since LGTV is an attenuated member of the TBEV complex, we next examined whether LRP also interacts with TBEV NS5. Indeed, LRP successfully pulled down TBEV-NS5 in co-immunoprecipitation assays (Fig 3E), indicating a conserved mechanism across related flaviviruses. Collectively, these results demonstrate that LRP is a proviral factor that enhances LGTV replication by interacting with viral replication machinery.

### LRP facilitates LGTV replication by promoting LDs degradation

**Fig 4:**
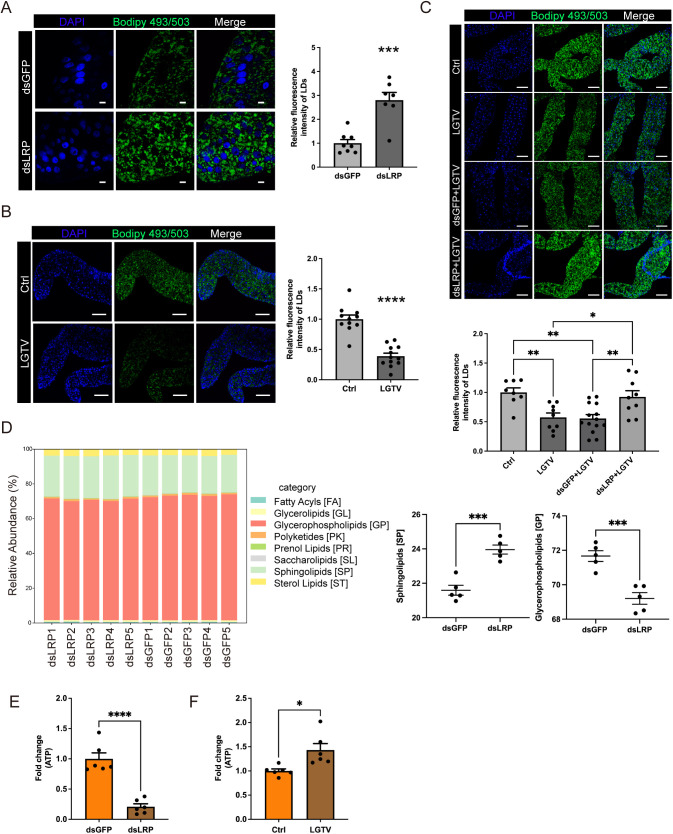
LRP-mediated LD catabolism facilitates LGTV replication. (**A**–**C**) Immunofluorescence staining of LDs in midguts of dsRNA-treated ticks fed on uninfected mice for two days (A), ticks fed on LGTV-infected mice for two days (B), and dsRNA-treated ticks fed on LGTV-infected mice for two days (C). The dsGFP-injected nymphs (*A*) and the nymphs bitten DMEM-injected A6 mice (*B*) were served as controls. The midguts were stained with Bodipy 493/503 (green). Nuclei were stained with DAPI (blue). Quantification of LDs fluorescence intensities was analyzed using ImageJ (right). (**D**) Lipid profiles following *LRP* knockdown. LRP deficiency significantly altered the midgut lipidome (right), specifically leading to a shift in sphingolipid and glycerophospholipid levels (left). (**E**, **F**) The total ATP levels during LRP knockdown (*E*) and LGTV infection (*F*) in F2D nymphs. All lipids showing significant changes are listed in S2 Table. (A) Scale bars, 500 μm. (B, C) Scale bars, 100 μm. Each dot represents individual image in (A–C), 10 midguts of nymphs in (*E*) and (F). Data are presented as mean ± SEM in (A) (*n* = 7–8), (B) (*n* = 11–12), (C) (*n* = 8–14), (D) (*n* = 5), (E) (*n* = 6), and (F) (*n* = 6). Significance was determined by Student *t* test in (A), (B), (D), (E), (F), and by one-way ANOVA in (C). * *p* < 0.05, ** *p* < 0.01, *** *p* < 0.001, **** *p* < 0.0001. The data underlying this figure are provided in S1 Raw Data.

LDLR family members mediate the uptake of cholesterol-carrying LDL particles and regulates lipid metabolism in mammals [25]. Although the LRP identified in *H. longicornis* lacks several domains found in mammalian LDLRs, it may still participate in lipid metabolic processes in ticks. To test this hypothesis, we knocked down *LRP* and analyzed LDs formation in tick midguts by BODIPY 493/503 staining. *LRP* knockdown resulted in a significant accumulation of LDs compared to dsGFP controls (Fig 4A), indicating that LRP promotes LDs turnover in ticks. Given that lipid metabolism is essential for viral replication [26,27], we next examined LDs dynamics during LGTV infection. Midguts from ticks that fed on LGTV-infected mice for 2 days were dissected and LDs levels were analyzed. As expected, LDs levels were markedly reduced in the midguts of ticks infected with LGTV compared to uninfected ones (Fig 4B). Notably, this virus-induced LDs reduction was abolished when *LRP* was knocked down (Fig 4C). These findings suggest that LGTV replication depends on LDs breakdown, a process mediated by LRP.

To identify the specific lipid species modulated by LRP, we performed a non-targeted lipidomic analysis of midguts from dsLRP- and dsGFP-treated nymphs. LRP deficiency significantly disrupted lipid homeostasis, with 9 lipid species reduced and 71 species accumulated (S2 Table). Specifically, sphingolipids were significantly increased, while glycerophospholipids were markedly reduced in *LRP*-knockdown midguts (Fig 4D). These results suggest that LRP-mediated pathways are vital for maintaining the lipid balance required for successful LGTV infection.

Considering that ATP derived from lipid metabolism is required for dengue virus replication [28], we investigated whether LRP-mediated viral inhibition occurs through the modulation of ATP production. We found that *LRP* knockdown led to a significant reduction in ATP levels in tick nymphs (Fig 4E). Conversely, LGTV infection significantly enhanced ATP generation (Fig 4F). Collectively, these data demonstrate that LRP-mediated LD mobilization is essential for maintaining bioenergetic and lipid homeostasis, which is critical for supporting LGTV replication.

### LRP promotes LDs degradation by lipophagy

**Fig 5:**
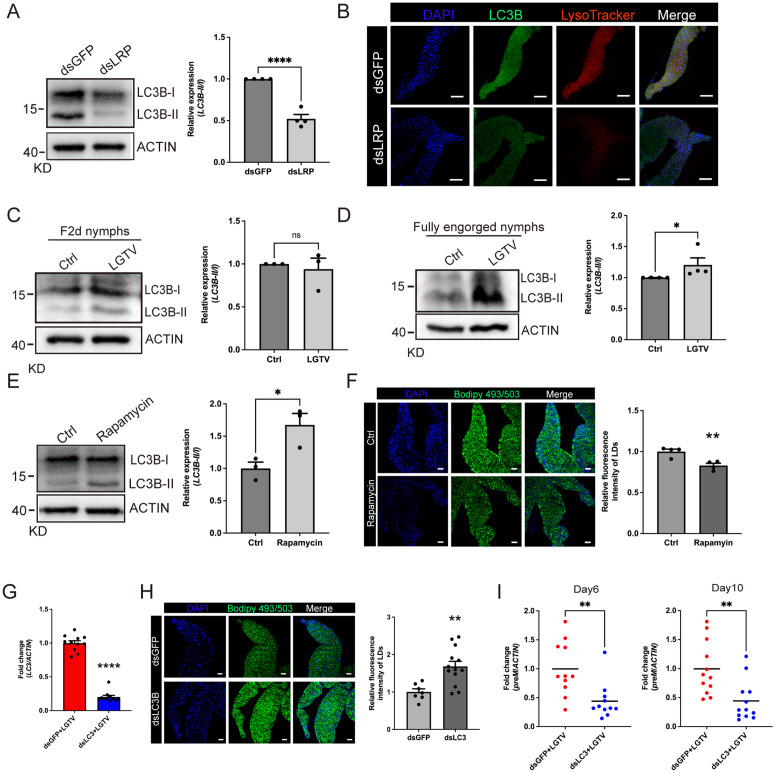
Lipophagy is essential for LGTV replication. (**A**, **C**, **D**, **E**) Western blot (left) and quantification analysis (right) of LC3B in dsGFP/dsLRP-treated nymphs (A), LGTV-infected nymphs for feeding for 2 days (C) and fully engorged (D), and rapamycin-treated ticks (E). ACTIN was used as internal control. (B) Whole-mount staining of LC3B (green) and lysosome (red) in midgut of dsRNA-treated ticks. Nuclei were stained with DAPI (blue). Scale bars, 100 μm. (**F**, **H**) Whole-mount staining of LDs in midgut of rapamycin-treated (*F*) or dsLC3B-treated (*H*) ticks via Bodipy 493/503 (green). Nuclei were stained with DAPI (blue). Quantification of LDs fluorescence intensity was analyzed using ImageJ (right). Scale bars, 50 μm. (**G**, **I**) Quantification of *LC3* knocking down efficiency (*G*) and *preM* levels (*I*) by qPCR in dsLC3- and dsGFP-treated nymphs. Each dot represents 2 pooled nymphs in (G), (I), an individual image in (F), (H), and an independent replicates in (A), (C), (D), (E). Data are presented as mean ± SEM in (G) (*n* = 11), (H) (*n* = 7–13). Horizontal lines represent the mean in (I) (*n* = 11–12). Significance was determined by Student *t* test in (*F*–*I*), and Mann–Whitney test in (A, C–E). * *p* < 0.05, ** *p* < 0.01, *** *p* < 0.001, **** *p* < 0.0001. The data underlying this figure are provided in S1 Data. The uncropped blots are included in S1 Raw Images.

Autophagy plays a role in LDs metabolism [28]. To determine whether LRP promotes LD degradation via autophagy, we silenced *LRP* expression and examined the levels of the microtubule-associated protein light chain 3 (LC3), a widely used marker of autophagy. Knockdown of *LRP* resulted in a significant reduction in LC3 levels (Fig 5A). Consistently, fluorescence signals for lysosomes and LC3B were markedly decreased in the midguts of ticks treated with dsLRP compared to dsGFP controls (Fig 5B). Moreover, LC3 levels were significantly elevated in fully engorged ticks that fed on LGTV-infected mice, compared to uninfected controls (Fig 5C and 5D), suggesting that LGTV induces autophagy, potentially to promote LD degradation and facilitate viral replication.

To examine autophagy plays a role in LD regulation, we treated ticks with rapamycin, an autophagy activator. Rapamycin treatment significantly induced LC3 levels, suggesting the induction of autophagy (Fig 5E). At the same time, the LDs in the midguts were decreased significantly (Fig 5F). These results indicate that autophagy plays a role in LD degradation. To determine whether autophagy-mediated LD degradation is essential for LGTV infection, we inhibited autophagy by silencing *LC3* and assessed both LD accumulation and viral levels. As expected, *LC3* knockdown resulted in increased LD abundance and significantly reduced LGTV viremia (Fig 5G–5I).

Given the direct interaction between LRP and NS5, we next investigated whether this interaction contributes to the induction of autophagy. NS5-HA and LRP-Flag were co-transfected into BHK-21 cells, and autophagy was assessed by measuring LC3 levels. As expected, LRP expression alone promoted autophagy in BHK-21 cells, however, co-expression of LRP and NS5 did not further enhance LC3 levels (S6 Fig). We next speculated that the LRP-NS5 interaction might facilitate the colocalization of viral replication complexes with LDs. To test this, we knocked down *LRP* in ticks and allowed them to feed on LGTV-infected A6 mice for two days. Midguts were dissected and examined for the spatial distribution of viral double-stranded RNA and LDs. No co-localization was observed in either the *LRP* knockdown group or control group (S7 Fig). Given that LRP overexpression increases NS5 protein level (Fig 3D), it is highly possible that the interaction with LRP may first enhance the stability of NS5 or protect it from proteasomal degradation. Secondly, LRP-induced autophagy facilitates lipid catabolism, thereby providing energy for viral replication. However, the mechanistic significance of the LRP–NS5 interaction remains unclear and requires further investigation.

## Discussion

TBEV is an important tick-borne virus that causes permanent neurological complications or even death in human. However, how TBEV interacts with or evades tick immune system during its transmission in ticks remains poorly understood. In this study, using LGTV, a surrogate for TBEV, we demonstrate that LGTV exploits the tick JAK/STAT signaling pathway to facilitate its replication.

The JAK/STAT pathway was initially identified as a cytokine signaling mechanism in mammals and is now recognized as a critical component of antiviral defense in both vertebrates and invertebrates [10,29–33]. In humans, it orchestrates antiviral immunity by inducing the expression of numerous interferon-stimulated genes [34]. In *Drosophila*, activation of the JAK/STAT pathway by the cytokine Unpaired (UPD) protects against invertebrate iridescent virus 6 (IIV-6) infection [35]. In *Aedes aegypti*, RNAi-mediated silencing of the JAK/STAT components *Dome* or *Hop* significantly increases susceptibility to DENV [31]. In *Culex annulirostris*, knockdown of *Cullin* activates the JAK/STAT pathway and reduces West Nile virus replication [36]. In contrast to its generally antiviral role in insects and mammals, our findings reveal a pro-viral function for the JAK/STAT pathway in ticks. Knockdown of *Dome* or *STAT* significantly reduced LGTV infection in both *H. longicornis* and ISE6 cells. Notably, the function of this pathway in bacterial infections in ticks also appears to be context-dependent. For example, in *I. scapularis*, the JAK/STAT pathway exerts opposing effects on *A. phagocytophilum* and *B. burgdorferi* infections [12]. Given the considerable phylogenetic distance between insects, mammals, and ticks, it is possible that ticks have evolved a functionally distinct JAK/STAT pathway shaped by their unique physiology and diverse pathogens they encounter.

Beyond its role in pathogen infection, the JAK/STAT pathway also contributes to the developmental process in ticks. The knockdown of *Dome* and *STAT* impairs blood meal acquisition, development, and molting in *I. scapularis* through the crosstalk to Hedgehog and Notch-Delta pathways [12]. Here, we show that, this signaling pathway is also involved in the regulation of tick metabolism. Knockdown of *STAT* significantly inhibits the expression of LRP that is essential for LDs turnover. Although our data indicate that JAK/STAT pathway promote LD breakdown by regulating the expression of *LRP,* the underlying regulatory mechanism remains unclear. It is possible that STAT directly binds to the *LRP* promoter or interacts with other transcriptional regulators that initiate *LRP* expression. This possibility warrants further investigation. Moreover, it is important to acknowledge that the knockdown of *STAT* and *LRP* can exert broad effects on tick physiology, including reduced feeding success and developmental arrest. Such systemic impairment could, in theory, indirectly restrict viral replication by limiting the resources available to the pathogen. However, in this study, we employed a 2-day feeding window where blood meal acquisition remained consistent across all groups, suggesting that the initial reduction in viral load is a direct consequence of the disrupted molecular pathways. Furthermore, the use of microinjection allowed for the delivery of uniform viral titers, bypassing the influence of feeding-associated acquisition. While long-term viral persistence may be influenced by declining tick health, our data indicate that the LRP-mediated mechanism is a primary requirement for early viral replication.

LRP belongs to LDLR superfamily, which comprises structurally and functionally related proteins involved in diversity biological processes that impact health and disease [25]. In both mammals and insects, members of this family share a conserved architecture characterized by a large extracellular domain with ligand-binding motifs, a transmembrane domain, and an intracellular cytoplasmic tail [16,21]. In contrast, the LRP identified in our study is structurally and functionally distinct. First, *H. longicornis* LRP lacks a transmembrane domain and is localized intracellularly. Second, while mammalian LDLRs often serve as entry receptors for various viruses [15,16,21], *H. longicornis* LRP does not interact with the LGTV E protein and has no effect on viral binding or internalization. Instead, we found that tick LRP directly interacts with the NS5 protein of both LGTV and TBEV, a key factor in viral replication, and promotes LGTV replication. Our comparative genomic analysis identified similar LRP homologs lacking transmembrane domains in several *Ixodes* species, the principal vector of TBEV in Eurasia and North America. This conservation suggests that the recruitment of intracellular LRP to facilitate viral replication via lipophagy may not be unique to *Haemaphysalis*, but rather a more broadly distributed strategy among hard ticks. Further research is required to delineate the functional distinctions between classical membrane-bound LRPs and these atypical intracellular variants during tick-borne virus infection.

In insects, members of the LDLR superfamily, such as vitellogenin and lipophorin receptors, primarily mediate the endocytosis of yolk protein precursors and are essential for oocyte development [37]. Notably, vitellogenin and lipophorin also recognize and bind pathogen-associated molecular patterns, thereby influencing immune responses in various insect species [38]. These observations suggest that LDLR family members may also contribute to immune regulation and pathogen invasion. In this study, we demonstrate that LRP enhances viral infection in ticks by promoting lipophagy. Host-derived lipids are critical resources for flavivirus replication [28,39,40], and consistent with this, LGTV induces lipophagy in ticks. Silencing *LRP* disrupts LGTV-induced LD degradation, highlighting LRP’s role in facilitating lipophagy during infection. Our data suggest a model where LGTV hijacks the tick’s metabolic machinery to fuel its own replication. While many viruses stimulate de novo lipogenesis, our results with *ACS1* knockdown suggest that LGTV primarily exploits pre-existing host lipid stores. Through LRP-mediated lipophagy, these stored lipids are mobilized and likely funneled into fatty aicd β-oxidation, as evidenced by the significant increase in ATP levels during infection. As sphigolipids are vital for ZIKA virus infection [39], the lipidomic shift observed upon *LRP* knockdown, specifically increase of sphingolipids, further indicates that LGTV relies on tick lipophagy to obtain energy or nutrients necessary for viral replication membranes. However, the mechanism by which tick LRP promotes lipophagy remains unclear. Although we showed interaction of LRP and NS5 increases NS5 protein levels, the functional basis for this interaction is still not fully understood. Further investigation is needed to elucidate the precise role of this interaction.

LGTV used to serve as a live-attenuated vaccine candidate for TBEV due to its naturally reduced virulence in humans, though its discontinuation due to rare cases of encephalitis, underscoring the complexity of its pathogenicity [41,42]. Our findings reveal that LGTV NS5 interacts with LRP to induce lipophagy, a mechanism that provides a metabolic advantage for viral replication in ticks. This reliance on specific host-mediated pathways may offer a mechanistic explanation for LGTV attenuation. Specifically, if the binding affinity of LGTV NS5 for mammalian orthologs of LRP is lower, or if mammalian lipophagy is less conducive to flavivirus replication than in ticks, this could restrict the virus’s ability to reach high titers in the human central nervous system. Such “host-specialization” could explain the limited neurovirulence of LGTV relative to the more robust and versatile replication strategies employed by TBEV.

## Materials and methods

### Ethics statement

All animal experiments were performed in accordance with the guidelines of the animal care and use of Fudan University and were approved by the Experimental Animal Welfare and Ethics Committee of Fudan University (2023JS059).

### Viruses

The LGTV strain TP21 used in this study was kindly provided by professor Zhenhua Zheng, Wuhan Institute of Virology, Chinese Academy of Sciences, and was handled under BSL2. The LGTV were passaged in BHK-21 cells by infecting them at a MOI of 0.1. Supernatants were harvested when cell mortality reached 70%. Virus stock was then aliquoted and stored at −80 °C. Viral titers were assessed by plaque formation assay as previously described [7].

### Cells

BHK-21 (baby hamster kidney cell line) and HEK-293T (human embryonic kidney cell line) cells were maintained in Dulbecco’s minimal essential medium (DMEM, WISENT, 319-005-CL) supplemented with 10% heat-inactivated fetal bovine serum (FBS, VivaCell BI, C04001-500) and 1% penicillin-streptomycin (PS, Gibco, 15140122). Cells were maintained at 37 °C in a 5% CO_2_ incubator. The ISE6 tick cell line from the embryo of *I. scapularis* was grown in L15B300 medium supplemented with 5% FBS (Gibco, 10091148), 10% tryptose phosphate broth (BD, 260300), and 1% lipoprotein (MP, 191476). Cells were maintained at 30 °C without CO_2_. BHK-21 and ISE6 cells were kindly provided by professor Zhenhua Zheng, Wuhan Institute of Virology, Chinese Academy of Sciences.

### Mice

Six- to eight-week-old C57BL/6 mice deficient in the type I interferon (IFN) receptors (A6 mice) were used for the virus infection studies and were kindly provided by professor Yang Li, Institute of Zoology, Chinese Academy of Sciences. A6 mice were bred and maintained in a pathogen-free environment at the Fudan University Animal Facility. All animal experiments were approved by and performed under the guidelines of the Experimental Animal Welfare and Ethics Committee of Fudan University.

### Ticks

*H. longicornis* (parthenogenesis strain) was maintained in the insectary at Fudan University at 25 °C with 85% relative humidity and a 12 h/12 h light/dark photoperiod as previously described [43]. Each stage of *H. longicornis* was sustained by feeding on eight-week-old male BALB/C, which were purchased from JSJ-Lab in Shanghai.

### Gene silencing and LGTV infection in ticks

Detailed procedures for gene silencing in ticks have been described [44]. In brief, double-stranded RNA (dsRNA) was synthesized using the MEGAscript T7 transcription kit (Thermo, ABM13345) with primers listed in S3 Table. Ticks were microinjected with 20 ng of dsRNA through the anal pore. The inoculated ticks were then allowed to recover for 1 day under standard rearing conditions. The ticks were then allowed to feed on mice for two days. The efficiency of *Dome*, *STAT*, and *LRP* knockdown was determined by RT-qPCR. The target genes and corresponding primer sequences were shown in S3 Table.

For LGTV infection by microinjection, the dsRNA-treated nymphs were allowed to feed on BALB/c mice for two days. Following feeding, ticks were microinjected with 300 PFU LGTV via anal pore. Knockdown efficiency and viral RNA was assessed by quantitative real-time PCR (qRT-PCR) on day 6 and day 10.

For LGTV infection by A6 mouse feeding, six- to twelve-week-old A6 mice were intraperitoneally (i.p.) inoculated with 10 PFU of LGTV on day 0. On day 1, nymphs were injected with 20 ng of dsRNA via the anal pore. On day 2, 20 μL of blood was collected from each mouse, and viral load was quantified by qRT-PCR using primers targeting the *preM* gene. The dsRNA-treated nymphs were then allowed to feed on mice with comparable viral titers. Tick viral load was assessed at the indicated time points post-feeding by qRT-PCR.

### RNAi and LGTV infection in ISE6 cells

ISE6 cells were seeded in a 48-well plate at a density of 1 × 10^5^ cells/mL and incubated overnight at 30 °C. When reached 90% confluent, ISE6 cells were transfected with 500 ng dsRNA targeting *Dome, STAT*, or a non-targeting control dsGFP using the lipofectamine 3000 (Thermo, L3000015) according to the manufacturer’s instructions. At 24 h post-transfection, ISE6 cells were incubated with LGTV (MOI = 0.1) for 24 h. After infection, the cells were harvested and total RNA was extracted for knockdown efficiency and LGTV infection.

### RNA extraction and quantitative real-time PCR (qRT-PCR)

Ticks, cells, or mouse blood were collected at the indicated time points, and total RNA was isolated using the Trizol Reagent (AG, 21101) according to the manufacturer’s protocol. Reverse transcription of total RNA was performed using HifairⅢ 1st Strand cDNA Synthesis SuperMix (Yeasen, 11141ES60) according to the manufacturer’s protocol. Knockdown efficiency and viral genomes were then quantified by qPCR using Hifair qPCR SYBR Green Master Mix (Yeasen, 11201ES50). Gene expression was analyzed using the primers listed in S3 Table. The *H. longicornis ACTIN* was used as an internal reference gene. The knocking down efficiency of target genes in nymphs were determined using the 2^−ΔΔCt^ method [45]. Quantification of *preM* gene expression was calculated using a standard curve. All RT-qPCR reactions were performed on a CFX Connect Real-time System (BIO-RAD).

### Transcriptome analysis

The nymphs treated with dsGFP/dsSTAT and fed for two days were collected. Ten nymphs were pooled for one biological replicate. Four biological replicates of each treatment were used for transcriptome analysis. Total RNA was extracted using Trizol reagent. RNA sequencing was performed by Novogene (Beijing, China) using the IIIumina HiSeq platform. Trinity was used for transcriptome assembly [46], and seven databases were annotated for gene function, including NCBI non-redundant protein sequences (Nr), NCBI nucleotide sequences (Nt), protein family (Pfam), clusters of orthologous groups of proteins/euKaryotic ortholog groups (KOG/COG), a manually annotated and reviewed protein sequence database (Swiss-Prot), kyoto encyclopedia of genes and genomes (KEGG), and gene ontology (GO). The DESeq R package was used for differential expression analysis [47]. Genes with an adjusted *P*-value <0.05 and the log_2_ ratio change (the number of reads in dsSTAT ticks/ the number of reads in dsGFP ticks) >1 were considered to be significantly differentially regulated [44]. Heat maps were generated using RStudio software based on FPKM values [48] in which gene length bias was corrected.

### Co-immunoprecipitation

The ORFs of *STAT* (2,418 bp, PV815417) and *LRP* (2,934 bp, KAH9364317.1) genes were amplified by PCR from tick cDNA and inserted into a pCAGGS vector between EcoRI and XhoI sites using primers containing a C-terminal flag tag as listed in S3 Table, named pCAGGS-STAT and pCAGGS-LRP, respectively. All viral genes including E (1,497 bp), C (288 bp), NS1 (1,047 bp), NS2A (690 bp), NS2B (393 bp), NS3 (1,863 bp), NS4A (447 bp), NS4B (756 bp), NS5 (2,709 bp), used in this study were amplified and inserted into pCAGGS vector between EcoRI and XhoI sites using primers containing an N-terminal HA tag and listed in S3 Table, named pCAGGS-E, pCAGGS-C, pCAGGS-NS1, pCAGGS-NS2A, pCAGGS-NS2B, pCAGGS-NS3, pCAGGS-NS4A, pCAGGS-NS4B, pCAGGS-NS5. TBEV-NS5 was amplified from a TBEV-NS5-flag plasmid, which kindly provided by professor Zhenhua Zheng, Wuhan Institute of Virology, Chinese Academy of Sciences. The TBEV-NS5 construct was then inserted into the pCAGGS vector between EcoRI and XhoI sites using primers containing an N-terminal HA tag as listed in S3 Table, named pCAGGS-TBEV-NS5. Plasmids for transfection were prepared using the EndoFree Mini Plasmid Kit (TIANGEN, DP123).

Plasmids expressing viral genes and tick genes were co-transfected into HEK-293T cells with the lipo2000 (Thermo, 11668019) according to the manufacturer’s protocol. Forty-eight hours post-transfection, cell lysates were prepared and subjected to immunoprecipitation with anti-Flag or anti-HA antibodies. Immunoprecipitated proteins and 20% input lysates were analyzed by western blotting with the following primary antibodies: anti-Flag (Yeasen, 30505ES20), anti-HA (Yeasen, 3070ES20), anti-ACTIN (Abbkine, ABL1011), and anti-Envelope [7]. Horseradish-peroxidase-conjugated goat anti-mouse IgG (Abmart, M21001S) and goat anti-rabbit IgG (Abmart, M21002S) were used as secondary antibodies.

For the experiment investigating the effect of LRP overexpression on NS5, LRP-Flag and NS5-HA were co-transfected into BHK-21 cells at a mass ratio of 2:1, and whole cells were harvested for subsequent assays at 48 hours post-transfection.

### LGTV binding, entry, and replication assays in BHK-21 cells

BHK-21 cells were transfected with LRP-Flag and pCAGGS as described previously. At 48 h post-transfection, the BHK-21 cells were incubated with LGTV (MOI = 10) and adsorbed onto the cells for 1.5 h at 4 °C as described [49]. After adsorption, the cells were washed three times with pre-cold PBS to remove unbound virus particles. For the binding assay, the cells were directly lysed for RNA extraction. For the entry assay, pre-warmed FBS-free DMEM medium was added to the cell wells, and the plates were placed at 37 °C for 1.5 h. After internalized, the cells were washed with PBS to remove any virus that had not been internalized, and the cells were lysed for RNA extraction.

For LGTV replicon assay, 24 h post-transfection, cells were allowed to transfect LGTV replicon that lacks the structural genes *C*, *prM*, and *E*. After another 24 h, the cells were harvested and total RNA was extracted for LGTV infection using *NS5* expression.

### Immunofluorescence assay

For BHK-21 cells, the immunofluorescence assay was performed as described previously with slight modifications [7]. Cells were grown on 24-well chamber slides overnight. After transfection or infection, the slides were fixed with 4% PFA at room temperature (RT) for 15 min followed by permeabilized with PBS containing 0.1% Triton X-100 for 10 min. Cells were incubated with blocking buffer for 2 h and then were incubated with primary antibodies overnight at 4 °C. Antibodies used in this study included anti-HA (Proteintech, 81290–1-RR), anti-Flag and anti-E antibody [7]. After washing with PBST, the slides were incubated with secondary antibodies for 1 h. The secondary antibodies were Alexa Fluor 488-conjugated goat anti-mouse IgG (Thermo, A-11001), and Alexa Fluor 546-conjugated goat anti-rabbit IgG (Thermo, A-11071). The cells were stained with DAPI (Solarbio, C0065) and mounted on glass coverslips using mounting media (Sigma, F4680). Images were acquired with using a Nikon positive laser scanning confocal microscope. Pearson’s coefficient indexes between NS5 and LRP fluorescence intensities were measured and calculated by Image J.

For ticks, the midguts from dsRNA knockdown, LGTV infection, or rapamycin treatment (500 nM, 20nL) were dissected in PBS and fixed in a 1.5 mL Eppendorf tube containing 4% PFA for 1 h at RT. After washing with PBS, the midguts were permeabilized with PBS containing 0.1% Triton X-100 for 15 min and incubated with blocking buffer for 2 h, and then incubated with primary antibodies overnight at 4 °C. Antibodies used in this study included anti-LC3B (Abmart, T55992S), and mouse anti-dsRNA (J2) (English and Scientific Consulting Bt, 10010200). After washing with PBST, the slides were incubated with secondary antibodies for 1 h. The secondary antibodies were Alexa Fluor 488-conjugated goat anti-rabbit IgG (Thermo, 704060), Alexa Fluor 546-conjugated goat anti-mouse IgG (Thermo, A-11003), or Alexa Fluor 546-conjugated goat anti-rabbit IgG. The tissues were stained with DAPI and the slides were mounted on glass coverslips using mounting media (Sigma, F4680). Images were acquired with using a Nikon positive laser scanning confocal microscope. For LDs analysis, the midguts were stained with BODIPY 493/503 (MCE, HY-D1614) for 20 min at RT in the dark before staining with DAPI. For lysosome analysis, the midguts were stained with LysoTracker Deep Red (Thermo, L7528) for 10 min at RT in the dark before staining with DAPI. Mean fluorescence intensity from the whole midgut was measured and calculated by Image J.

### Lipidomic analysis

To characterize the lipidomic profiles, midguts were dissected from dsGFP- and dsLRP-treated nymphs after a two-day blood meal. For each biological replicate, 60 midguts were pooled to ensure sufficient biomass. A total of 5 independent biological replicates per group were subjected to non-targeted lipidomic analysis. Samples were analyzed via Liquid Chromatography-Mass Spectrometry (LC-MS/MS) by Aipudikang (Shanghai, China). Raw MS data were processed using Progenesis QI software (Waters), and lipid identification was performed by searching against the Lipid-MAPS database.

### Quantification of cellular ATP levels

Tick midguts were collected at the indicated time points post-feeding. For each biological replicate, 10 midguts were pooled. A total of 6 independent biological replicates per experimental group were processed. Intracellular ATP levels were quantified using an Enhanced ATP Assay Kit (Beyotime, S0027) following the manufacturer’s protocols. Luminescence was measured using a multimode microplate reader, and data are expressed as relative ATP levels compared to the control groups.

### Autophagy assay

Nymphs were microinjected with dsRNA, 20 nL 500 nM rapamycin/PBS through anal pore and subsequently allowed to feed on BALB/c for 2 days. Nymphs were allowed to bite DMEM/LGTV-infected A6 mice for 2 days or to fully engorged. Ticks were collected after feeding at indicated time.

Ten F2D ticks or four fully engorged ticks were pooled and homogenized in RIPA buffer containing protease and phosphatase inhibitor (Epizyme, GRF103). Lysates were then centrifuged at 16,000 g, 4 °C for 10 min to remove the pellet and the supernatant was collected. Western blot analyses were performed as previously described. The primary antibodies, anti-ACTIN and anti-LC3B, and the secondary antibodies, goat anti-rabbit IgG HRP, were used in this experiment.

### Statistical analysis

All analyses were performed using GraphPad Prism 8.0 software. The analyses of results were performed with a two-tailed unpaired Student *t* test for two groups, one-way ANOVA for three or more groups. Experiments were performed at least twice. *P* values of<0.05 were considered statistically significant.

## Supporting information

S1 Fig The influence of JAK/STAT pathway on tick feeding. (**A**, **B**) Engorgement weights of nymphs treated with dsGFP, dsDome, and dsSTAT. Each dot represents an individual tick. Horizontal lines represent the mean in (B) (*n* = 26–35). Significance was determined by one-way ANOVA. **** *p* < 0.0001. The data underlying this figure can be found in S1 Raw Data.
(TIF)

S2 Fig Interference of JAK/STAT pathway inhibits LGTV infection in ISE6 cells. (**A**) Schematic of the experimental design. ISE6 cells were transfected with 500 ng dsRNA targeting *Dome, STAT* of *I. scapularis*, or a non-targeting control dsGFP for 24 h, followed by infection with LGTV (MOI = 0.1). The cells were harvested for total RNA extraction. The knockdown efficiency and viral infection were analyzed at 24 hpi. (**B**, **D**) Silencing efficiency of *Dome* (B) and *STAT* (D) in ISE6 cells. (**C**, **E**) LGTV *preM* levels in dsDome (C) and dsSTAT (E) treated ISE6 cells. Each dot represents individual cell well in (B–E). Data are presented as mean ± SEM in (B) (*n* = 4) and (D) (*n* = 5). Horizontal lines represent the mean in (C) (*n* = 4) and (E) (*n* = 5). Significance was determined by Student *t* test in (B–E). * *p* < 0.05, ** *p* < 0.01, *** *p* < 0.001, **** *p* < 0.0001. The data underlying this figure can be found in S1 Raw Data.
(TIF)

S3 Fig Tick STAT has no direct interaction with LGTV NS proteins. Co-immunoprecipitation of STAT-Flag and NS-HA proteins, including NS1-HA (**A**), NS2A-HA (**B**), NS2B-HA (**C**), NS3-HA (**D**), NS4A-HA (**E**), NS4B-HA (**F**), and NS5-HA (**G**). The uncropped blots are included in S1 Raw Images.
(TIF)

S4 Fig The influence of STAT-regulated genes on LGTV infection. (**A**) Quantification of *LRP* gene levels by qPCR in dsDome and dsGFP. Knockdown efficiency and viral load in nymphs treated with dsACSl (**B**), dsVg (**C**), and dsPer (**D**). Nymphs treated with dsGFP were used as controls. Each dot represents 2 pooled nymphs in (B–D). Data are presented as mean ± SEM in (A) (*n* = 9–10) and (B–D, left) (*n* = 11–12). Horizontal lines represent the mean in (B–D, right) (*n* = 5–10). Significance was determined by Student *t* test in (B–D). ** *p* < 0.01, **** *p* < 0.0001. The data underlying this figure can be found in S1 Raw Data.
(TIF)

S5 Fig LRP does not influence LGTV entry process. (**A**) Comparative protein structures of human LDLR and tick LRP. Orthologs from *H. longicornis* (KAH9364317.1), *D. silvarum* (XP_049513863), *I. persulcatus* (KAG0432932, KAG0435715), *I. pacificus* (CAN8009861), and *I. scapularis* (XP_042143231) are shown. (**B**) Co-immunoprecipitation of LRP and E protein. (**C**) Co-localization of LRP with E in BHK-21 cells. Scale bar, 50 μm. (**D**) The *preM* levels of bound and internalized LGTV in LRP-transfected BHK-21 cells. Cells transfected with pCAGGS were used as controls. Each dot represents individual cell well in (D). Data are presented as mean ± SEM in (D) (*n* = 5–6). Significance was determined by Student *t* test in (D). The data underlying this figure can be found in S1 Raw Data. The uncropped blots are included in S1 Raw Images.
(TIF)

S6 Fig Effects of LRP and NS5 overexpression on autophagy. Western analysis of LC3B in BHK-21 cells expressing NS5 and LRP. The pCAGGS vector, NS5, LRP were served as controls. ACTIN was used as internal control. The data underlying this figure can be found in S1 Raw Data. The uncropped blots are included in S1 Raw Images.
(TIF)

S7 Fig The interaction between LRP and NS5 has no influence on the localization of virus and LDs. Whole-mount staining of dsRNA (red) and LDs (green) in midgut of dsRNA-treated and LGTV-infected ticks. Nuclei were stained with DAPI (blue). Scale bars, 100 μm.
(TIF)

S1 Table List of differentially regulated genes in dsGFP and dsSTAT-treated nymphs. (XLSX)

S2 Table List of significantly altered lipids in dsGFP- and dsLRP-treated nymphs. (XLSX)

S3 Table Primers used in this study. (XLSX)

S1 Raw Images All the uncropped western blot images contained in the manuscript. Uncropped western blot images corresponding to Fig 2H (**A**), Fig 3B left (**B**), Fig 3B right (**C**), Fig 3D (**D**), Fig 3E (**E**), Fig 5A (**F**), Fig 5C (**G**), Fig 5D (**H**), Fig 5E (**I**), S3 Fig (**J**–**P**), S5 Fig (**Q**), S6 Fig (**R**).
(PDF)

S1 Raw Data All the raw data contained in the manuscript. (XLSX)

S1 Data Western analysis of LC3B in BHK-21 cells expressing NS5 and LRP. (XLSX)

## Abbreviations

ALSV: Alongshan virus
BSL-3: biosafety level 3
dsRNA: double-stranded RNA
EGF: epidermal growth factor
GO: gene ontology
IFN: interferon
KEGG: kyoto encyclopedia of genes and genomes
KFDV: Kyasanur Forest disease virus
LBD: ligand binding domain
LC3: light chain 3
LDs: lipid droplets
LGTV: Langat virus
LRP: lipoprotein receptor-related protein
MOI: multiplicity of infection
NS: non-structural
POWV: Powassan virus
qRT-PCR: quantitative real-time PCR
RT: room temperature
SMART: simple modular architecture research tool
TBEV: tick-borne encephalitis virus

## References

[1] Moraga-Fernández A, Muñoz-Hernández C, Sánchez-Sánchez M, Fernández de Mera IG, de la Fuente J. Exploring the diversity of tick-borne pathogens: The case of bacteria (*Anaplasma*, *Rickettsia*, *Coxiella* and *Borrelia*) protozoa (*Babesia* and *Theileria*) and viruses (*Orthonairovirus*, tick-borne encephalitis virus and louping ill virus) in the European continent. Vet Microbiol. 2023;286:109892. doi: 10.1016/j.vetmic.2023.109892 37866329

[2] Shi J, Hu Z, Deng F, Shen S. Tick-borne viruses. Virol Sin. 2018;33(1):21–43. doi: 10.1007/s12250-018-0019-0 29536246 PMC5866268

[3] Pulkkinen LIA, Butcher SJ, Anastasina M. Tick-borne encephalitis virus: a structural view. Viruses. 2018;10(7):350. doi: 10.3390/v10070350 29958443 PMC6071267

[4] Yoshii K. Epidemiology and pathological mechanisms of tick-borne encephalitis. J Vet Med Sci. 2019;81(3):343–7. doi: 10.1292/jvms.18-0373 30674746 PMC6451894

[5] Ličková M, Fumačová Havlíková S, Sláviková M, Klempa B. Alimentary infections by tick-borne encephalitis virus. Viruses. 2021;14(1):56. doi: 10.3390/v14010056 35062261 PMC8779402

[6] Feng T, Tong H, Zhang Q, Ming Z, Song Z, Zhou X, et al. Targeting Haemaphysalis longicornis serpin to prevent tick feeding and pathogen transmission. Insect Sci. 2024;31(3):694–706. doi: 10.1111/1744-7917.13260 37635449

[7] Xu Y, Wang J. The vector competence of Asian longhorned ticks in langat virus transmission. Viruses. 2024;16(2). doi: 10.3390/v16020304 38400079 PMC10893034

[8] Fogaça AC, Sousa G, Pavanelo DB, Esteves E, Martins LA, Urbanová V, et al. Tick immune system: what is known, the interconnections, the gaps, and the challenges. Front Immunol. 2021;12:628054. doi: 10.3389/fimmu.2021.628054 33737931 PMC7962413

[9] Hajdušek O, Síma R, Ayllón N, Jalovecká M, Perner J, de la Fuente J, et al. Interaction of the tick immune system with transmitted pathogens. Front Cell Infect Microbiol. 2013;3:26. doi: 10.3389/fcimb.2013.00026 23875177 PMC3712896

[10] Liu L, Dai J, Zhao YO, Narasimhan S, Yang Y, Zhang L, et al. Ixodes scapularis JAK-STAT pathway regulates tick antimicrobial peptides, thereby controlling the agent of human granulocytic anaplasmosis. J Infect Dis. 2012;206(8):1233–41. doi: 10.1093/infdis/jis484 22859824 PMC3448968

[11] Narasimhan S, Rajeevan N, Liu L, Zhao YO, Heisig J, Pan J, et al. Gut microbiota of the tick vector Ixodes scapularis modulate colonization of the Lyme disease spirochete. Cell Host Microbe. 2014;15(1):58–71. doi: 10.1016/j.chom.2013.12.001 24439898 PMC3905459

[12] Rana VS, Kitsou C, Dutta S, Ronzetti MH, Zhang M, Bernard Q, et al. Dome1-JAK-STAT signaling between parasite and host integrates vector immunity and development. Science. 2023;379(6628):eabl3837. doi: 10.1126/science.abl3837 36634189 PMC10122270

[13] Ngono AE, Shresta S. Immune response to dengue and zika. Annu Rev Immunol. 2018;36:279–308. doi: 10.1146/annurev-immunol-042617-053142 29345964 PMC5910217

[14] Ezeonwumelu IJ, Garcia-Vidal E, Ballana E. JAK-STAT pathway: a novel target to tackle viral infections. Viruses. 2021;13(12). doi: 10.3390/v13122379 34960648 PMC8704679

[15] Monteil VM, Wright SC, Dyczynski M, Kellner MJ, Appelberg S, Platzer SW, et al. Crimean-Congo haemorrhagic fever virus uses LDLR to bind and enter host cells. Nat Microbiol. 2024;9(6):1499–512. doi: 10.1038/s41564-024-01672-3 38548922 PMC11153131

[16] Xu Z-S, Du W-T, Wang S-Y, Wang M-Y, Yang Y-N, Li Y-H, et al. LDLR is an entry receptor for Crimean-Congo hemorrhagic fever virus. Cell Res. 2024;34(2):140–50. doi: 10.1038/s41422-023-00917-w 38182887 PMC10837205

[17] Chen L, Zhang J, Xu W, Chen J, Tang Y, Xiong S, et al. Cholesterol-rich lysosomes induced by respiratory syncytial virus promote viral replication by blocking autophagy flux. Nat Commun. 2024;15(1):6311. doi: 10.1038/s41467-024-50711-4 39060258 PMC11282085

[18] Kung Y-A, Chiang H-J, Li M-L, Gong Y-N, Chiu H-P, Hung C-T, et al. Acyl-coenzyme A synthetase long-chain family member 4 is involved in viral replication organelle formation and facilitates virus replication via ferroptosis. mBio. 2022;13(1):e0271721. doi: 10.1128/mbio.02717-21 35038927 PMC8764547

[19] Clark LE, Clark SA, Lin C, Liu J, Coscia A, Nabel KG, et al. VLDLR and ApoER2 are receptors for multiple alphaviruses. Nature. 2022;602(7897):475–80. doi: 10.1038/s41586-021-04326-0 34929721 PMC8808280

[20] Ma H, Kim AS, Kafai NM, Earnest JT, Shah AP, Case JB, et al. LDLRAD3 is a receptor for Venezuelan equine encephalitis virus. Nature. 2020;588(7837):308–14. doi: 10.1038/s41586-020-2915-3 33208938 PMC7769003

[21] Zhai X, Li X, Veit M, Wang N, Wang Y, Merits A, et al. LDLR is used as a cell entry receptor by multiple alphaviruses. Nat Commun. 2024;15(1):622. doi: 10.1038/s41467-024-44872-5 38245515 PMC10799924

[22] Pedersen NB, Wang S, Narimatsu Y, Yang Z, Halim A, Schjoldager KT-BG, et al. Low density lipoprotein receptor class A repeats are O-glycosylated in linker regions. J Biol Chem. 2014;289(25):17312–24. doi: 10.1074/jbc.M113.545053 24798328 PMC4067166

[23] Biswal M, Yao W, Lu J, Chen J, Morrison J, Hai R, et al. A conformational selection mechanism of flavivirus NS5 for species-specific STAT2 inhibition. Commun Biol. 2024;7(1):76. doi: 10.1038/s42003-024-05768-8 38195857 PMC10776582

[24] Grant A, Ponia SS, Tripathi S, Balasubramaniam V, Miorin L, Sourisseau M, et al. Zika virus targets human STAT2 to inhibit type I interferon signaling. Cell Host Microbe. 2016;19(6):882–90. doi: 10.1016/j.chom.2016.05.009 27212660 PMC4900918

[25] Willnow TE. The low-density lipoprotein receptor gene family: multiple roles in lipid metabolism. J Mol Med (Berl). 1999;77(3):306–15. doi: 10.1007/s001090050356 10090593

[26] Ma Y-X, Chai Y-J, Han Y-Q, Zhao S-B, Yang G-Y, Wang J, et al. Pseudorabies virus upregulates low-density lipoprotein receptors to facilitate viral entry. J Virol. 2024;98(1):e0166423. doi: 10.1128/jvi.01664-23 38054618 PMC10804996

[27] Lange PT, Lagunoff M, Tarakanova VL. Chewing the fat: the conserved ability of DNA viruses to hijack cellular lipid metabolism. Viruses. 2019;11(2):119. doi: 10.3390/v11020119 30699959 PMC6409581

[28] Heaton NS, Randall G. Dengue virus-induced autophagy regulates lipid metabolism. Cell Host Microbe. 2010;8(5):422–32. doi: 10.1016/j.chom.2010.10.006 21075353 PMC3026642

[29] Dupuis S, Jouanguy E, Al-Hajjar S, Fieschi C, Al-Mohsen IZ, Al-Jumaah S, et al. Impaired response to interferon-alpha/beta and lethal viral disease in human STAT1 deficiency. Nat Genet. 2003;33(3):388–91. doi: 10.1038/ng1097 12590259

[30] Dostert C, Jouanguy E, Irving P, Troxler L, Galiana-Arnoux D, Hetru C, et al. The Jak-STAT signaling pathway is required but not sufficient for the antiviral response of *Drosophila*. Nat Immunol. 2005;6(9):946–53. doi: 10.1038/ni1237 16086017

[31] Souza-Neto JA, Sim S, Dimopoulos G. An evolutionary conserved function of the JAK-STAT pathway in anti-dengue defense. Proc Natl Acad Sci U S A. 2009;106(42):17841–6. doi: 10.1073/pnas.0905006106 19805194 PMC2764916

[32] Boutros M, Agaisse H, Perrimon N. Sequential activation of signaling pathways during innate immune responses in *Drosophila*. Dev Cell. 2002;3(5):711–22. doi: 10.1016/s1534-5807(02)00325-8 12431377

[33] Paradkar PN, Trinidad L, Voysey R, Duchemin J-B, Walker PJ. Secreted Vago restricts West Nile virus infection in Culex mosquito cells by activating the Jak-STAT pathway. Proc Natl Acad Sci U S A. 2012;109(46):18915–20. doi: 10.1073/pnas.1205231109 23027947 PMC3503207

[34] MacMicking JD. Interferon-inducible effector mechanisms in cell-autonomous immunity. Nat Rev Immunol. 2012;12(5):367–82. doi: 10.1038/nri3210 22531325 PMC4150610

[35] West C, Silverman N. p38b and JAK-STAT signaling protect against Invertebrate iridescent virus 6 infection in *Drosophila*. PLoS Pathog. 2018;14(5):e1007020. doi: 10.1371/journal.ppat.1007020 29746571 PMC5963806

[36] Paradkar PN, Duchemin J-B, Rodriguez-Andres J, Trinidad L, Walker PJ. Cullin4 is pro-viral during West Nile virus infection of *Culex* mosquitoes. PLoS Pathog. 2015;11(9):e1005143. doi: 10.1371/journal.ppat.1005143 26325027 PMC4556628

[37] Tufail M, Takeda M. Insect vitellogenin/lipophorin receptors: molecular structures, role in oogenesis, and regulatory mechanisms. J Insect Physiol. 2009;55(2):87–103. doi: 10.1016/j.jinsphys.2008.11.007 19071131

[38] Leyria J, Fruttero LL, Paglione PA, Canavoso LE. How insects balance reproductive output and immune investment. Insects. 2025;16(3). doi: 10.3390/insects16030311 40266843 PMC11943238

[39] Leier HC, Weinstein JB, Kyle JE, Lee J-Y, Bramer LM, Stratton KG, et al. A global lipid map defines a network essential for Zika virus replication. Nat Commun. 2020;11(1):3652. doi: 10.1038/s41467-020-17433-9 32694525 PMC7374707

[40] Zhang J, Lan Y, Li MY, Lamers MM, Fusade-Boyer M, Klemm E, et al. Flaviviruses exploit the lipid droplet protein AUP1 to trigger lipophagy and drive virus production. Cell Host Microbe. 2018;23(6):819-831.e5. doi: 10.1016/j.chom.2018.05.005 29902443

[41] Asghar N, Jaafar R, Valko A, Merinder O, Ljungberg K, Lindqvist CM, et al. Development of Langat virus infectious clones as a platform for live-attenuated tick-borne encephalitis vaccine. Npj Viruses. 2025;3(1):44. doi: 10.1038/s44298-025-00129-6 40410304 PMC12102213

[42] Gritsun TS, Lashkevich VA, Gould EA. Tick-borne encephalitis. Antiviral Res. 2003;57(1–2):129–46. doi: 10.1016/s0166-3542(02)00206-1 12615309

[43] Wang M, Zhu D, Dai J, Zhong Z, Zhang Y, Wang J. Tissue localization and variation of major symbionts in *Haemaphysalis longicornis*, *Rhipicephalus haemaphysaloides*, and *Dermacentor silvarum* in China. Appl Environ Microbiol. 2018;84(10):e00029-18. doi: 10.1128/AEM.00029-18 29523550 PMC5930374

[44] Zhong Z, Zhong T, Peng Y, Zhou X, Wang Z, Tang H, et al. Symbiont-regulated serotonin biosynthesis modulates tick feeding activity. Cell Host Microbe. 2021;29(10):1545-1557.e4. doi: 10.1016/j.chom.2021.08.011 34525331

[45] Livak KJ, Schmittgen TD. Analysis of relative gene expression data using real-time quantitative PCR and the 2−ΔΔCT method. Methods. 2001;25(4):402–8. doi: 10.1006/meth.2001.1262 11846609

[46] Grabherr MG, Haas BJ, Yassour M, Levin JZ, Thompson DA, Amit I, et al. Full-length transcriptome assembly from RNA-Seq data without a reference genome. Nat Biotechnol. 2011;29(7):644–52. doi: 10.1038/nbt.1883 21572440 PMC3571712

[47] Love MI, Huber W, Anders S. Moderated estimation of fold change and dispersion for RNA-seq data with DESeq2. Genome Biol. 2014;15(12):550. doi: 10.1186/s13059-014-0550-8 25516281 PMC4302049

[48] Trapnell C, Williams BA, Pertea G, Mortazavi A, Kwan G, van Baren MJ, et al. Transcript assembly and quantification by RNA-Seq reveals unannotated transcripts and isoform switching during cell differentiation. Nat Biotechnol. 2010;28(5):511–5. doi: 10.1038/nbt.1621 20436464 PMC3146043

[49] Fan W, Mar KB, Sari L, Gaszek IK, Cheng Q, Evers BM, et al. TRIM7 inhibits enterovirus replication and promotes emergence of a viral variant with increased pathogenicity. Cell. 2021;184(13):3410-3425.e17. doi: 10.1016/j.cell.2021.04.047 34062120 PMC8276836

