# Supplementary figures and images for: A non-canonical JAK/STAT pathway promotes viral replication through the lipoprotein receptor-related protein in ticks
Source: PLoS Biol. 2026 May 21;24(5):e3003797. doi: 10.1371/journal.pbio.3003797 (PMC13193390; doi:10.1371/journal.pbio.3003797)

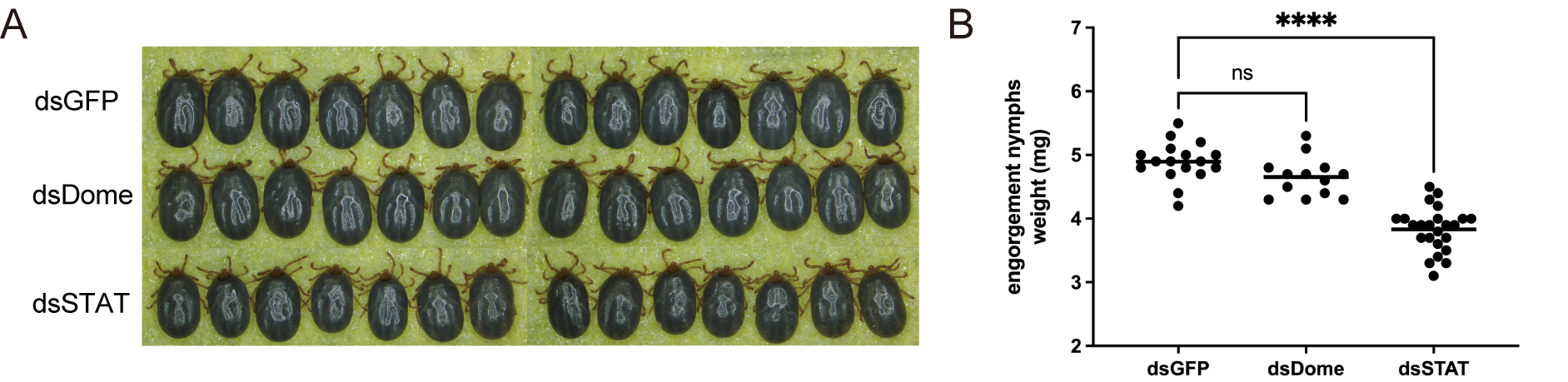

Supplement: S1 Fig — (A, B) Engorgement weights of nymphs treated with dsGFP, dsDome, and dsSTAT. Each dot represents an individual tick. Horizontal lines represent the mean in (B) (n = 26–35). Significance was determined by one-way ANOVA. **** p < 0.0001. The data underlying this figure can be found in S1 Raw Data. (TIF) [file pbio.3003797.s001.tif]

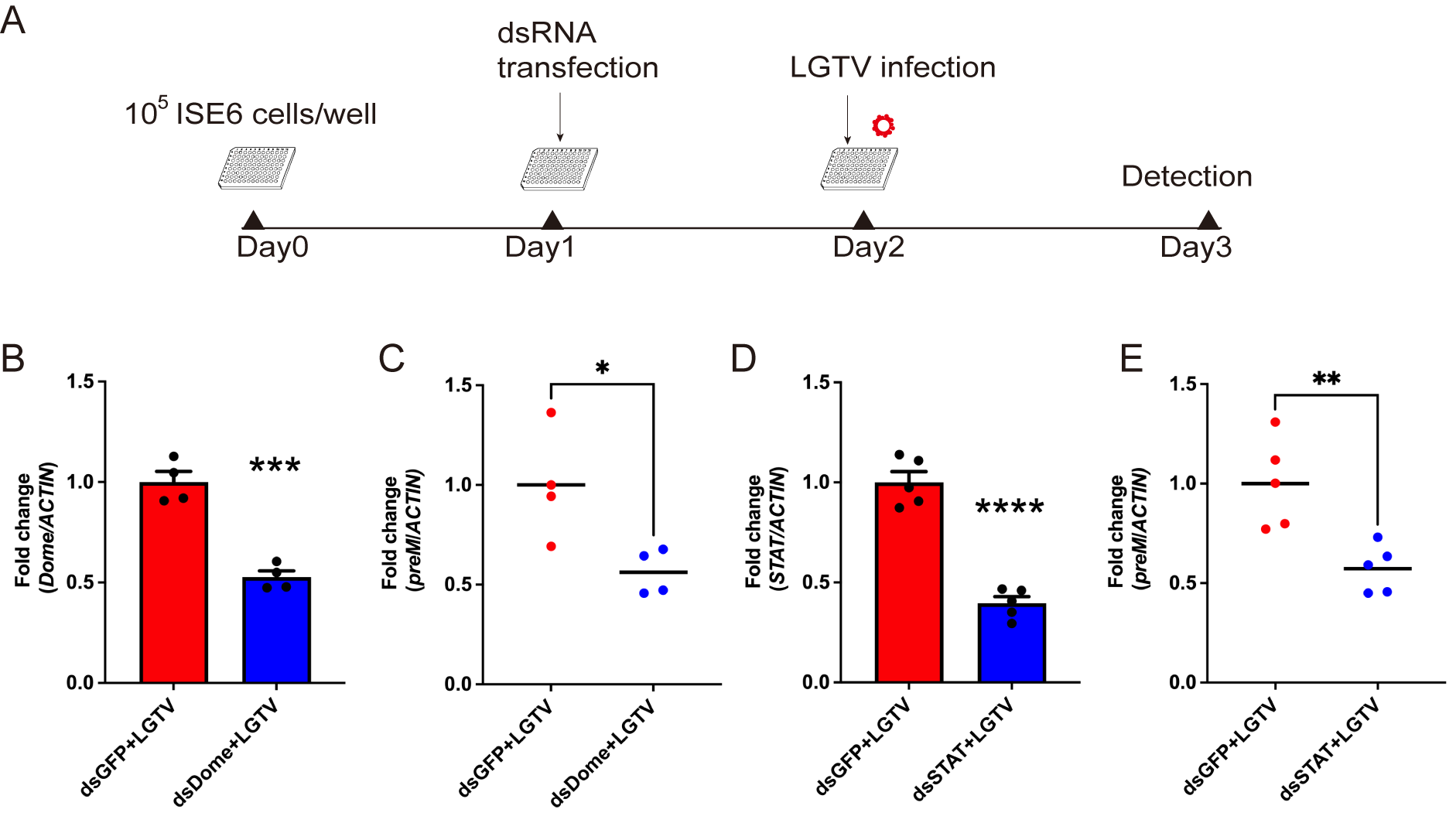

Supplement: S2 Fig — (A) Schematic of the experimental design. ISE6 cells were transfected with 500 ng dsRNA targeting Dome, STAT of I. scapularis, or a non-targeting control dsGFP for 24 h, followed by infection with LGTV (MOI = 0.1). The cells were harvested for total RNA extraction. The knockdown efficiency and viral infection were analyzed at 24 hpi. (B, D) Silencing efficiency of Dome (B) and STAT (D) in ISE6 cells. (C, E) LGTV preM levels in dsDome (C) and dsSTAT (E) treated ISE6 cells. Each dot represents individual cell well in (B–E). Data are presented as mean ± SEM in (B) (n = 4) and (D) (n = 5). Horizontal lines represent the mean in (C) (n = 4) and (E) (n = 5). Significance was determined by Student t test in (B–E). * p < 0.05, ** p < 0.01, *** p < 0.001, **** p < 0.0001. The data underlying this figure can be found in S1 Raw Data. (TIF) [file pbio.3003797.s002.tif]

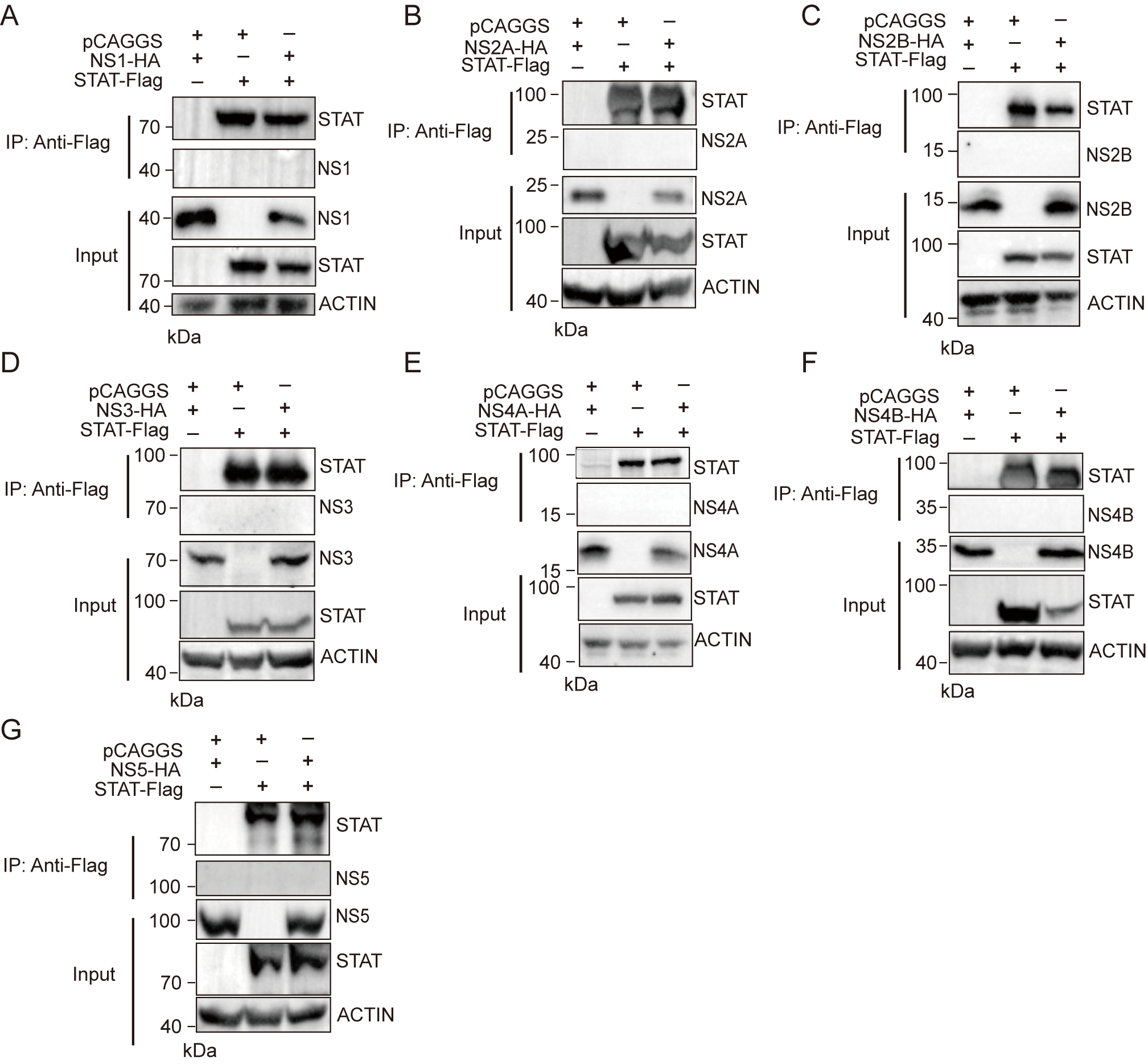

Supplement: S3 Fig — Co-immunoprecipitation of STAT-Flag and NS-HA proteins, including NS1-HA (A), NS2A-HA (B), NS2B-HA (C), NS3-HA (D), NS4A-HA (E), NS4B-HA (F), and NS5-HA (G). The uncropped blots are included in S1 Raw Images. (TIF) [file pbio.3003797.s003.tif]

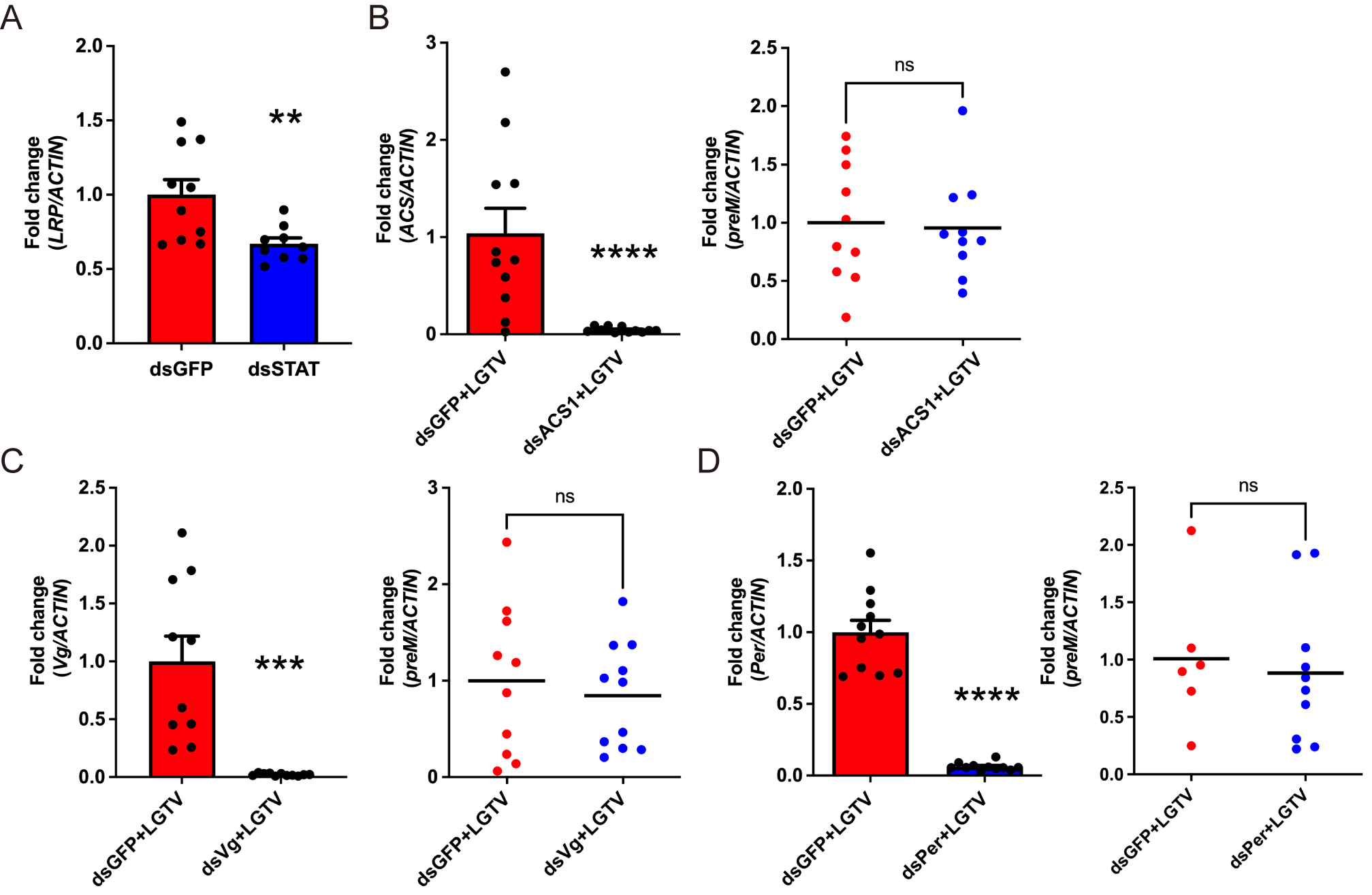

Supplement: S4 Fig — (A) Quantification of LRP gene levels by qPCR in dsDome and dsGFP. Knockdown efficiency and viral load in nymphs treated with dsACSl (B), dsVg (C), and dsPer (D). Nymphs treated with dsGFP were used as controls. Each dot represents 2 pooled nymphs in (B–D). Data are presented as mean ± SEM in (A) (n = 9–10) and (B–D, left) (n = 11–12). Horizontal lines represent the mean in (B–D, right) (n = 5–10). Significance was determined by Student t test in (B–D). ** p < 0.01, **** p < 0.0001. The data underlying this figure can be found in S1 Raw Data. (TIF) [file pbio.3003797.s004.tif]

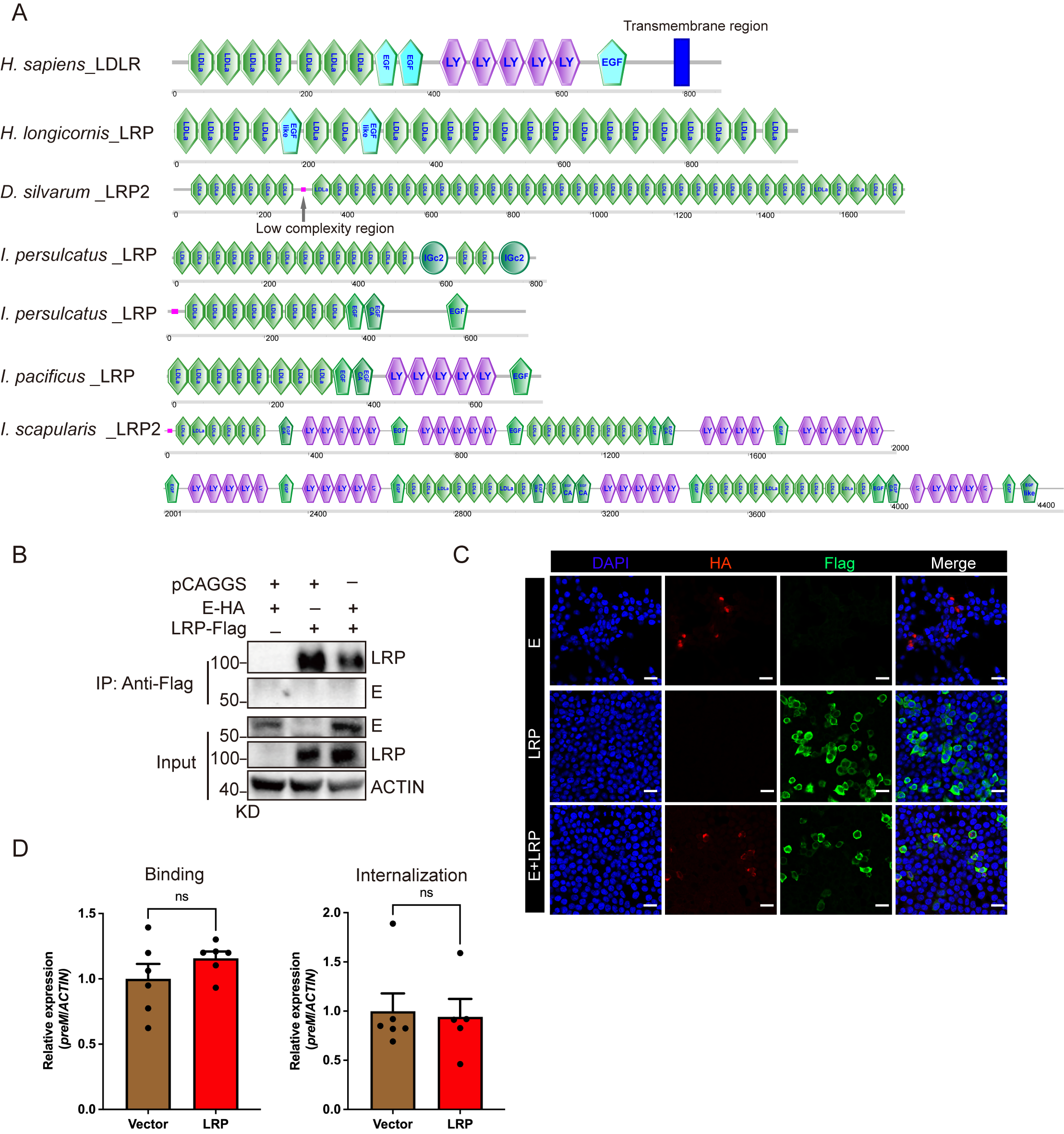

Supplement: S5 Fig — (A) Comparative protein structures of human LDLR and tick LRP. Orthologs from H. longicornis (KAH9364317.1), D. silvarum (XP_049513863), I. persulcatus (KAG0432932, KAG0435715), I. pacificus (CAN8009861), and I. scapularis (XP_042143231) are shown. (B) Co-immunoprecipitation of LRP and E protein. (C) Co-localization of LRP with E in BHK-21 cells. Scale bar, 50 μm. (D) The preM levels of bound and internalized LGTV in LRP-transfected BHK-21 cells. Cells transfected with pCAGGS were used as controls. Each dot represents individual cell well in (D). Data are presented as mean ± SEM in (D) (n = 5–6). Significance was determined by Student t test in (D). The data underlying this figure can be found in S1 Raw Data. The uncropped blots are included in S1 Raw Images. (TIF) [file pbio.3003797.s005.tif]

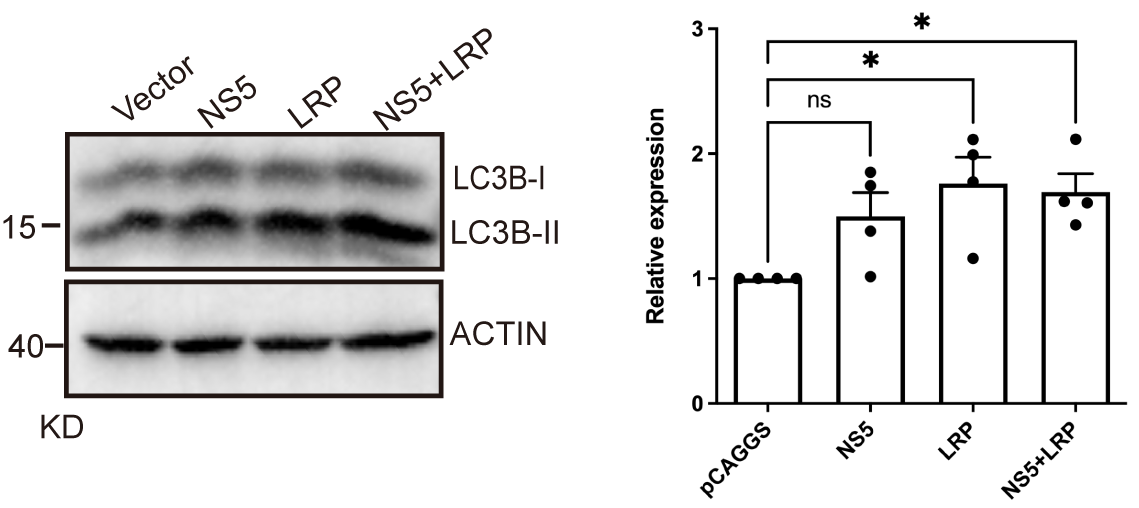

Supplement: S6 Fig — Western analysis of LC3B in BHK-21 cells expressing NS5 and LRP. The pCAGGS vector, NS5, LRP were served as controls. ACTIN was used as internal control. The data underlying this figure can be found in S1 Raw Data. The uncropped blots are included in S1 Raw Images. (TIF) [file pbio.3003797.s006.tif]

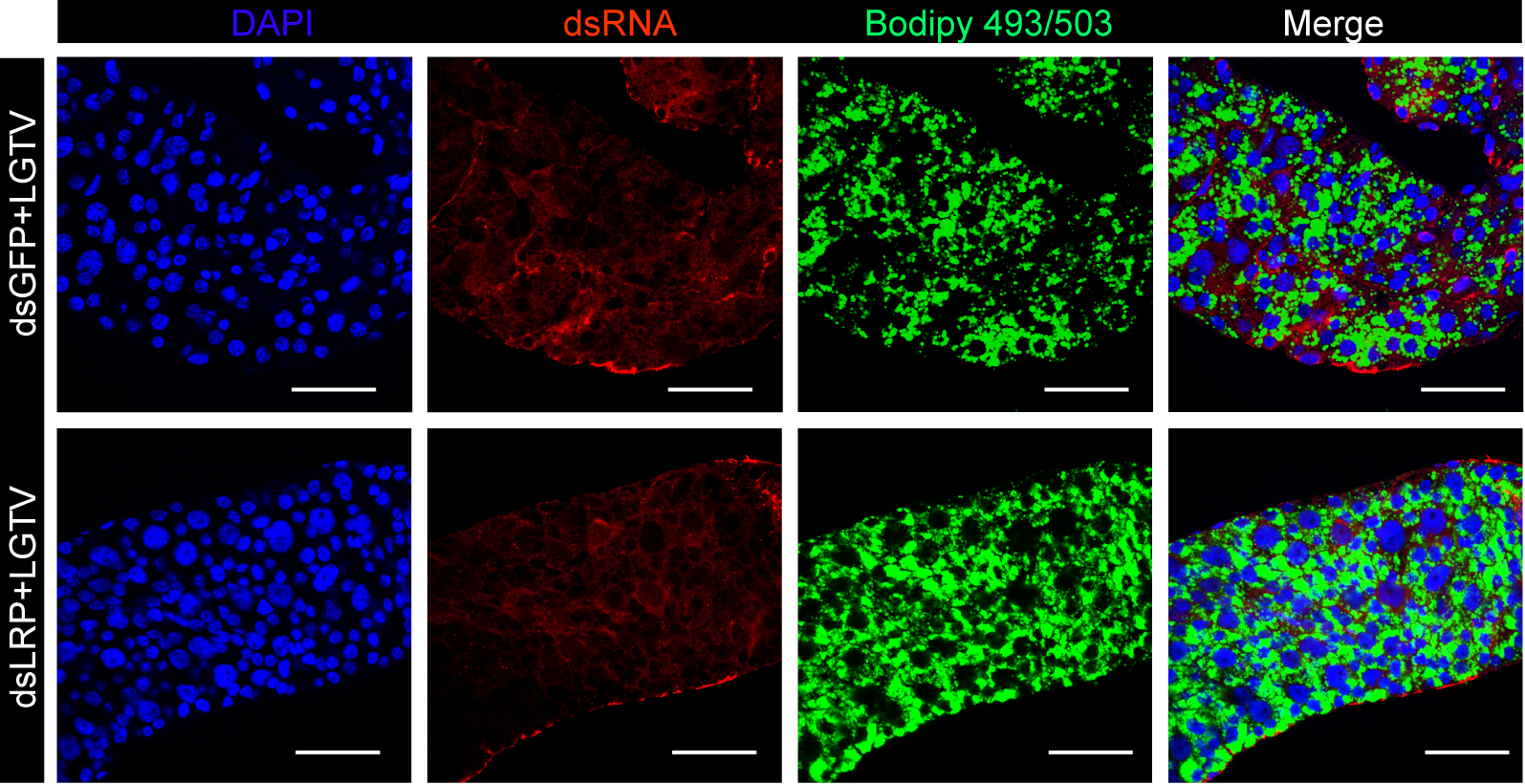

Supplement: S7 Fig — Whole-mount staining of dsRNA (red) and LDs (green) in midgut of dsRNA-treated and LGTV-infected ticks. Nuclei were stained with DAPI (blue). Scale bars, 100 μm. (TIF) [file pbio.3003797.s007.tif]

**A**  
**Fig 2H**

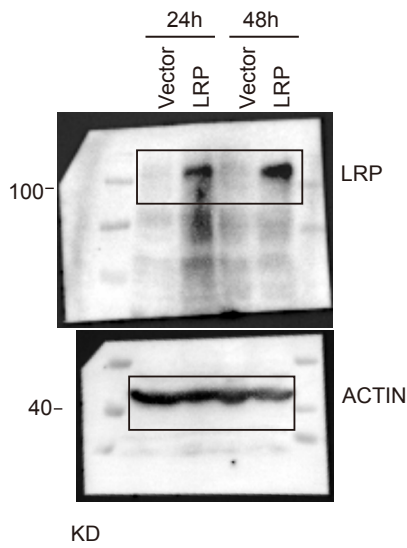

**B**  
**Fig 3B (left)**

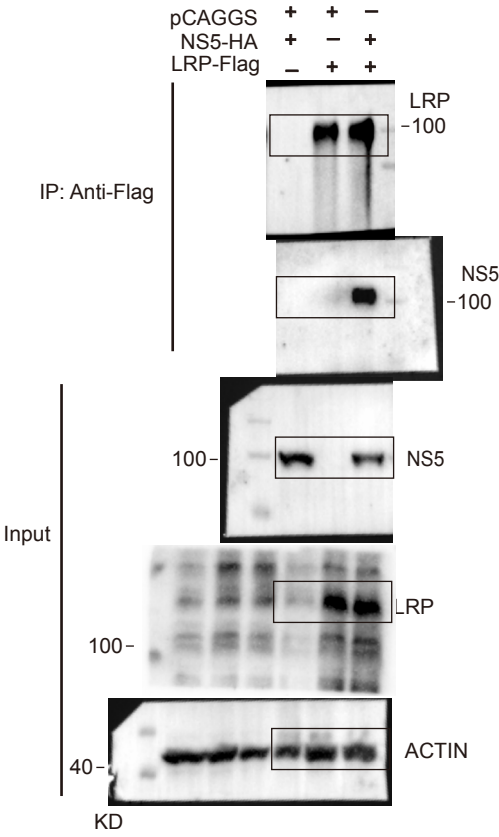

**C**  
**Fig 3B (right)**

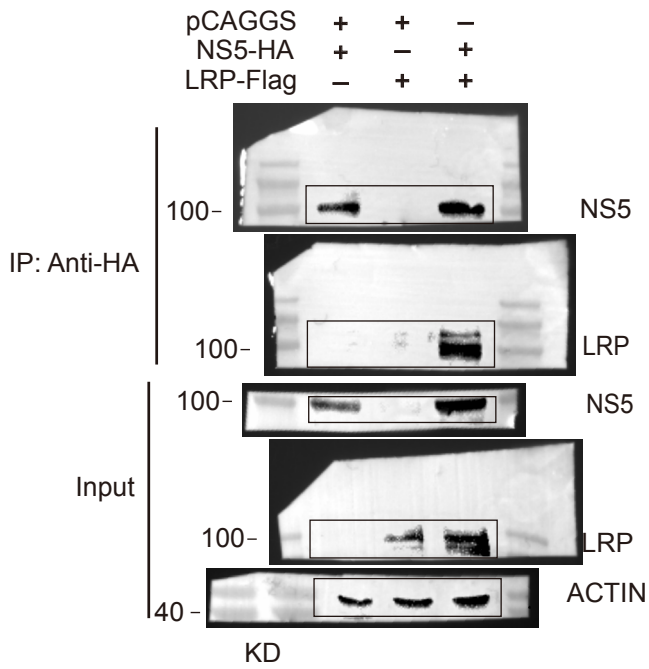

**D**  
**Fig 3D**

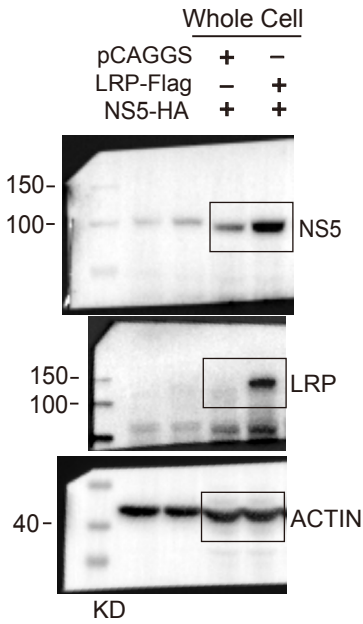

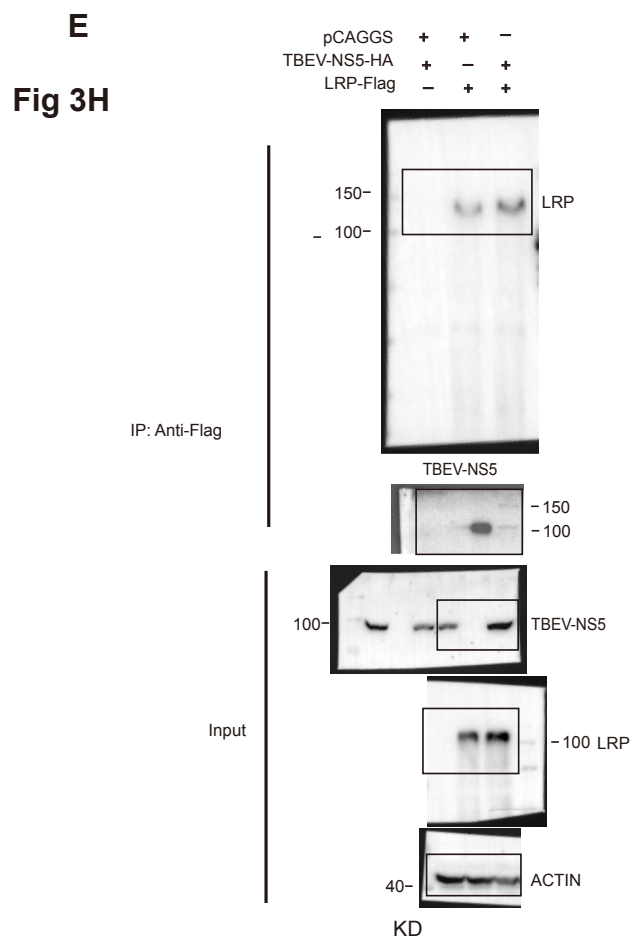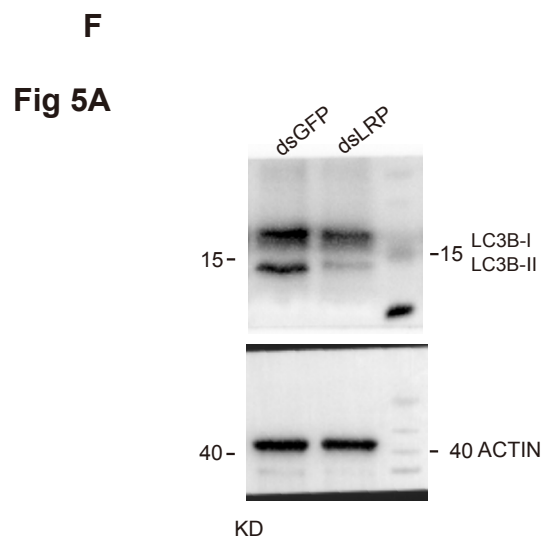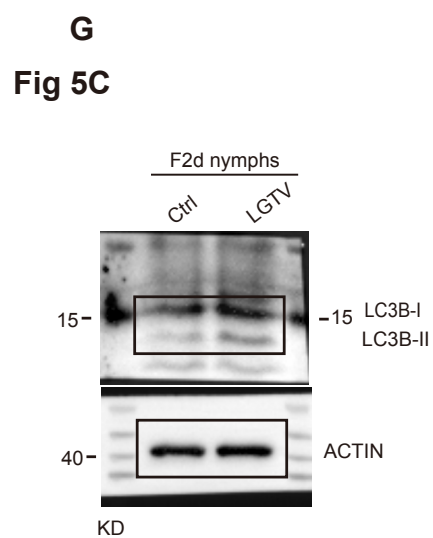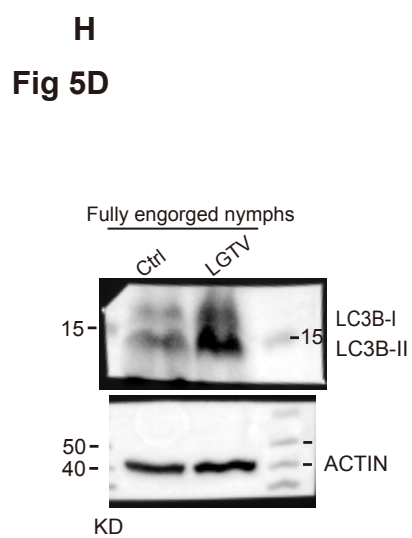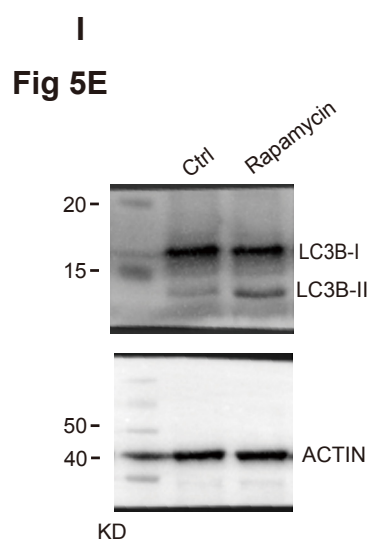

**J**  
**S3 Fig A**

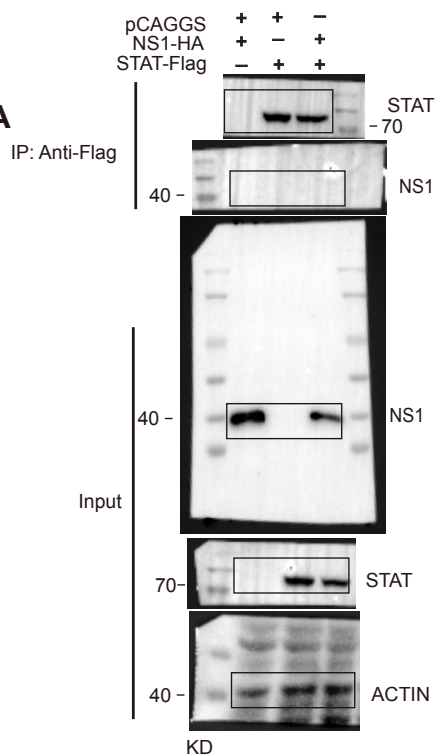

**K**  
**S3 Fig B**

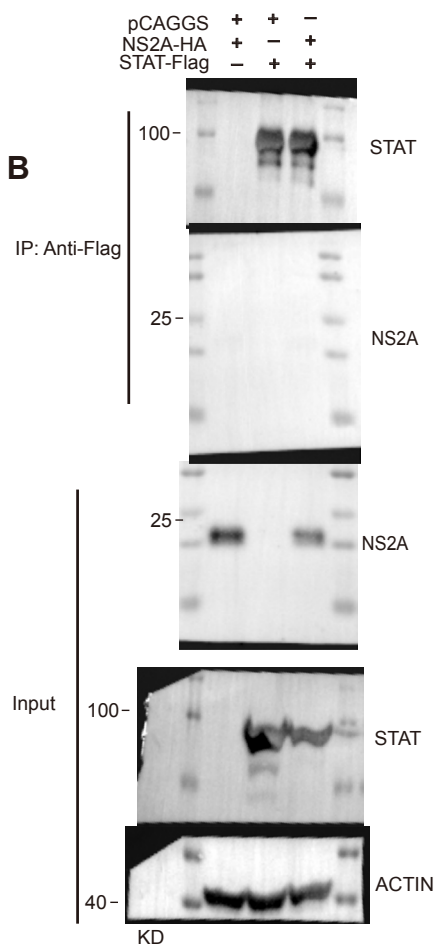

**L**  
**S3 Fig C**

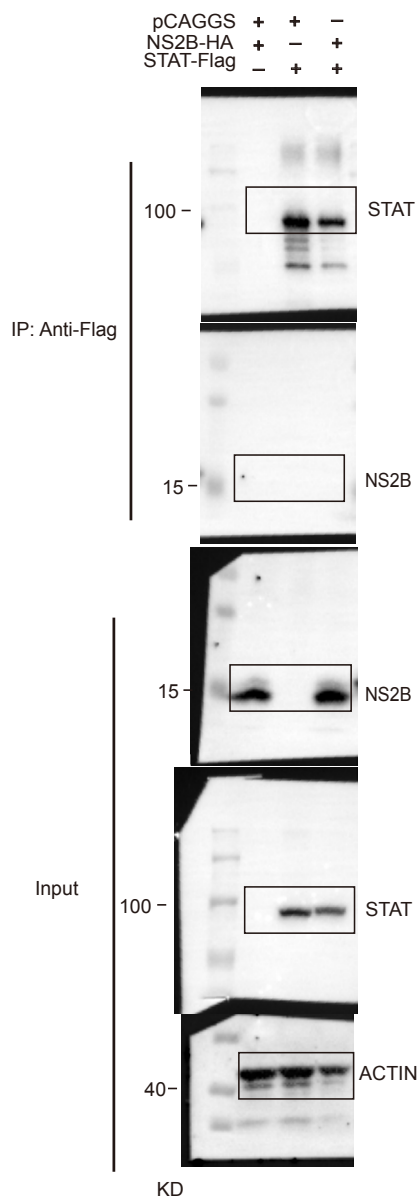

**M**  
**S3 Fig D**

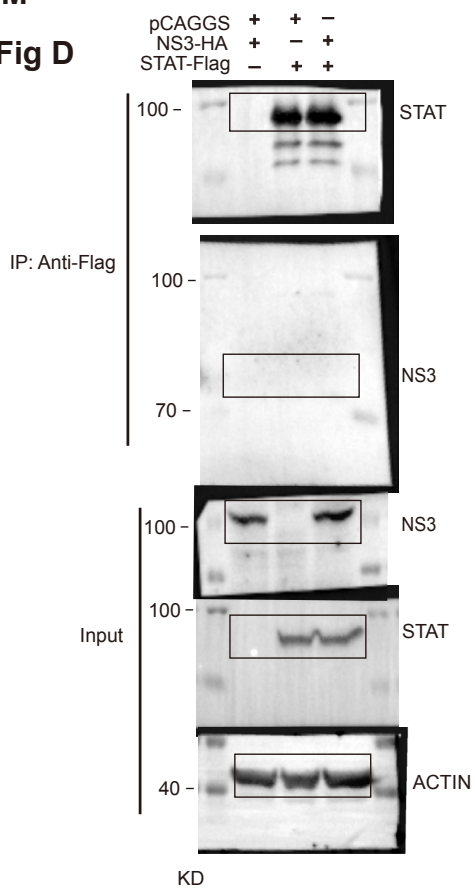

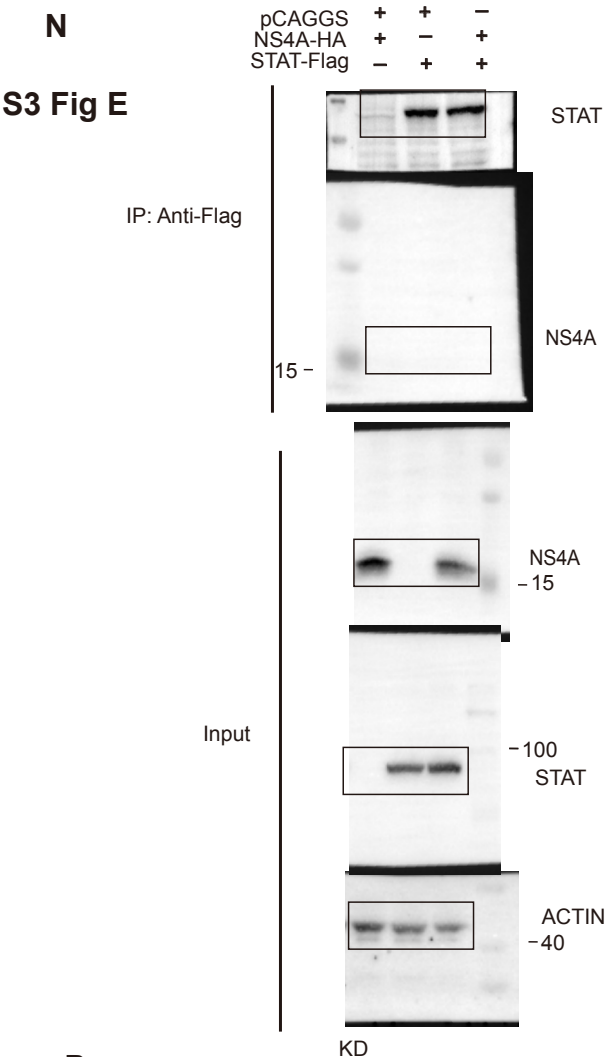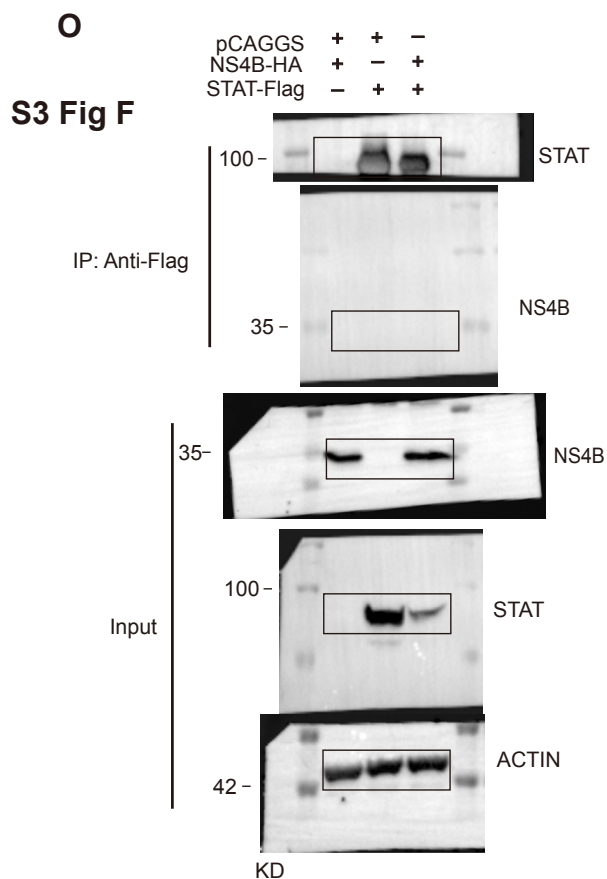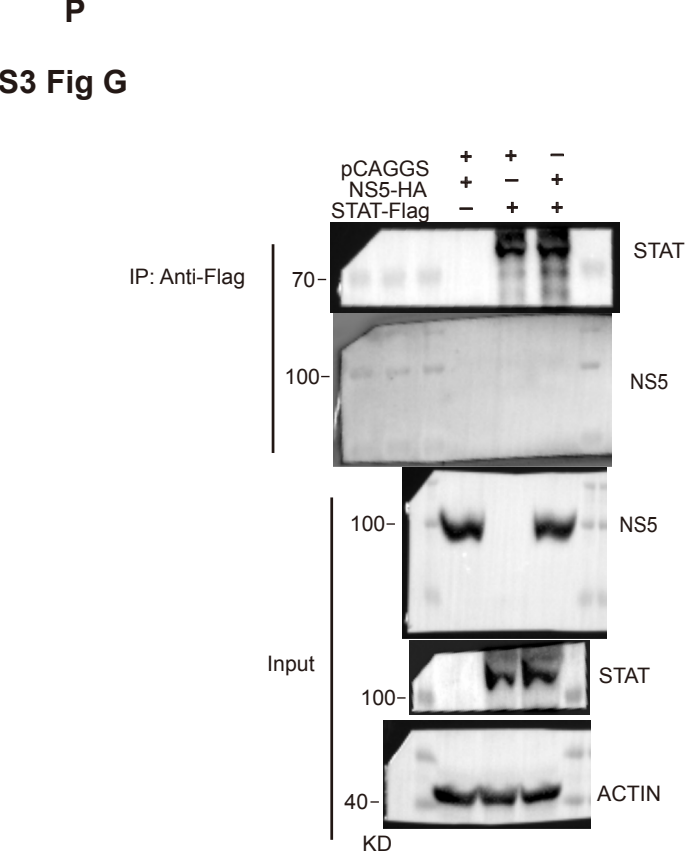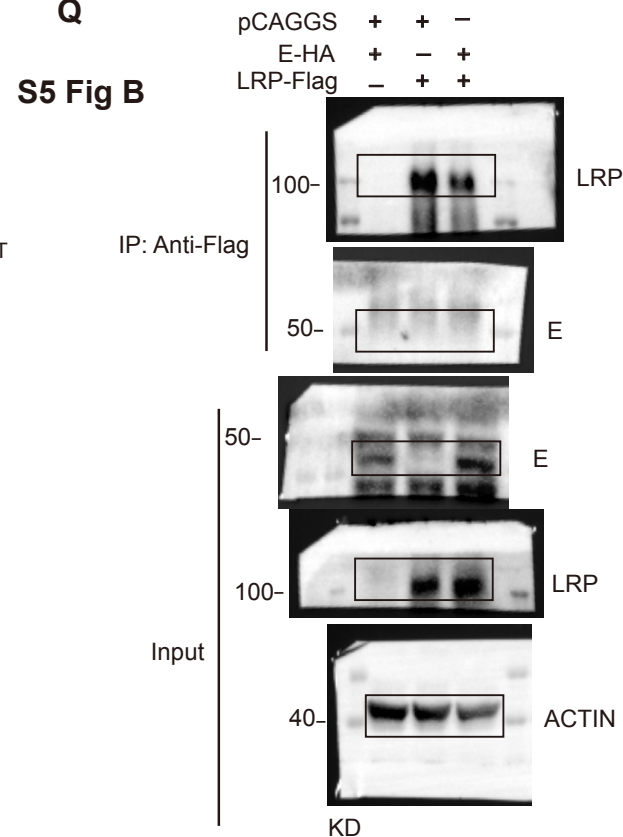

R

S6 Fig

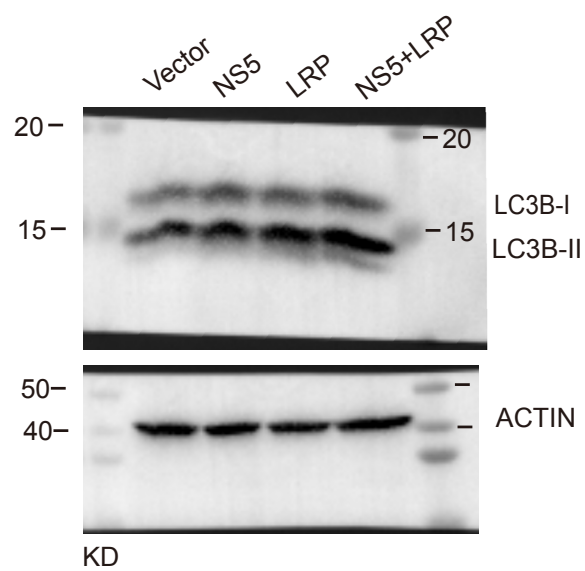

Supplement: S1 Raw Images — Uncropped western blot images corresponding to Fig 2H (A), Fig 3B left (B), Fig 3B right (C), Fig 3D (D), Fig 3E (E), Fig 5A (F), Fig 5C (G), Fig 5D (H), Fig 5E (I), S3 Fig (J–P), S5 Fig (Q), S6 Fig (R). (PDF) [file pbio.3003797.s011.pdf]
